# Distributed desalination using solar energy: A technoeconomic framework to decarbonize nontraditional water treatment

**DOI:** 10.1016/j.isci.2023.105966

**Published:** 2023-01-13

**Authors:** Akanksha K. Menon, Mingxin Jia, Sumanjeet Kaur, Chris Dames, Ravi S. Prasher

**Affiliations:** 1 George W. Woodruff School of Mechanical Engineering, Georgia Institute of Technology, Atlanta, GA 30332, USA; 2 Energy Storage & Distributed Resources Division, Lawrence Berkeley National Laboratory, Berkeley, CA 94720, USA; 3 Department of Mechanical Engineering, University of California, Berkeley, Berkeley, CA 94720, USA

**Keywords:** energy resources, engineering, energy sustainability, energy modeling, water resources engineering

## Abstract

Desalination using renewable energy offers a route to transform our incumbent linear consumption model to a circular one. This transition will also shift desalination from large-scale centralized coastal facilities toward modular distributed inland plants. This new scale of desalination can be satisfied using solar energy to decarbonize water production, but additional considerations, such as storage and inland brine management, become important. Here, we evaluate the levelized cost of water for 16 solar desalination system configurations at 2 different salinities. For fossil fuel-driven plants, we find that zero-liquid discharge is economically favorable to inland brine disposal. For renewable desalination, we discover that solar-thermal energy is superior to photovoltaics due to low thermal storage cost and that energy storage, despite being expensive, outperforms water storage as the latter has a low utilization factor. The analysis also yields a promising outlook for solar desalination by 2030 as solar generation and storage costs decrease.

## Highlights

• Fossil fuel-driven zero-liquid discharge is more economical than inland brine disposal
• Solar-thermal energy is better suited to power solar desalination than photovoltaics
• Energy storage is a more economically favorable storage option than water storage
• With reductions in solar and storage costs, solar desalination will become competitive

## Graphical abstract

**Figure:**
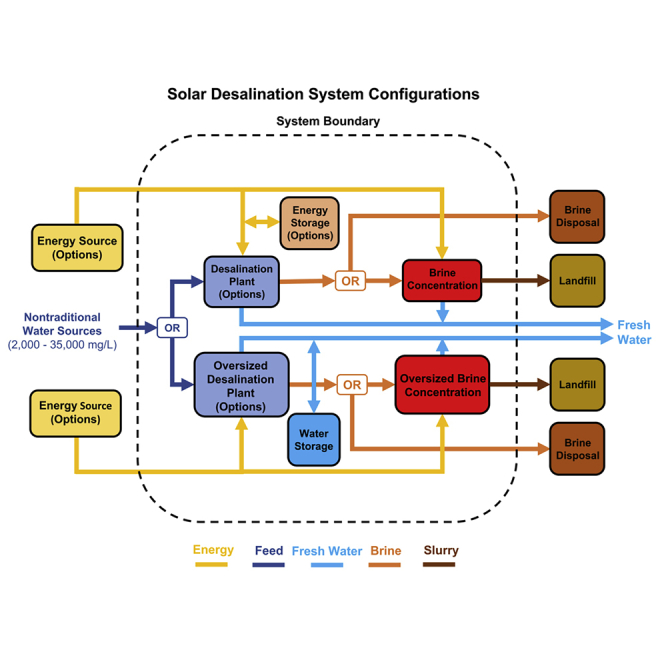

## Introduction

**Figure 1:**
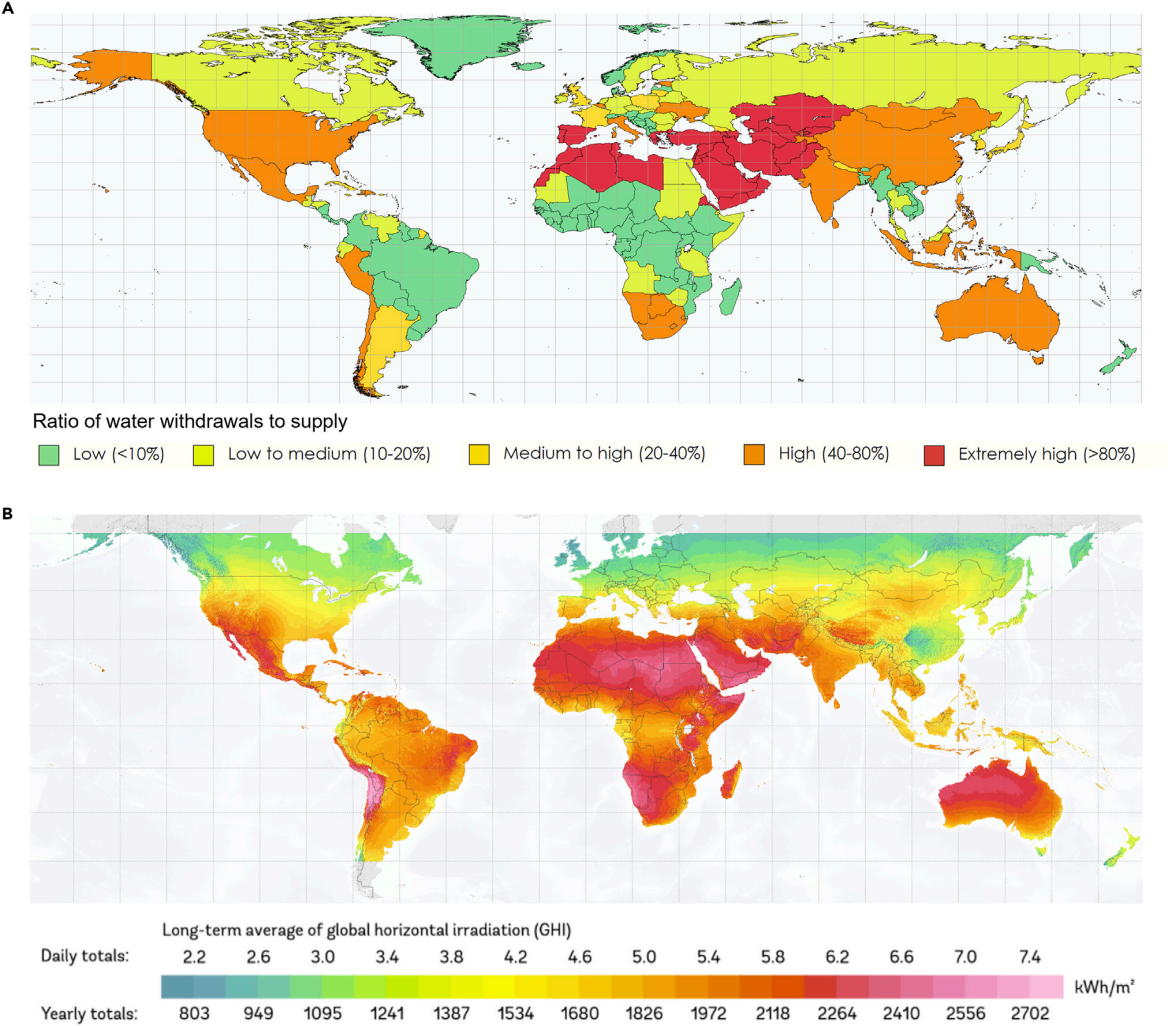
Potential for solar-driven desalination (A) Projected water stress by country in 2040 (adapted from the World Resources Institute).^12^ (B) Solar resource in terms of the daily and annual global horizontal irradiation (adapted from The World Bank Group and Solargis).^13^ The strong correlation between the two maps indicates the potential for solar-driven desalination across different regions of the world.

Global population growth and economic development have led to rising water demands, which when coupled with dwindling freshwater reserves due to climate change, is exacerbating water scarcity.^1^^,^^2^ Projections indicate that over half the global population will experience severe water stress by the end of this decade, thus necessitating the use of desalination technologies to close the gap between water demand and supply.^3^^,^^4^ Although desalination has the potential to provide more reliable and climate-independent freshwater, its broader adoption is limited by the large energy footprint and associated treatment cost.^5^ Specifically, energy alone accounts for 30–50% of the total water cost, which is currently dominated by fossil fuel-driven purification of seawater.^6^^,^^7^ This carbon footprint is expected to become significant (1–10 kg CO_2_ per cubic meter of freshwater produced) as the global desalination capacity increases to 200 million m^3^/day by the end of this decade, thus suggesting a critical need to decarbonize water treatment and produce clean water sustainably.^8^^,^^9^^,^^10^ In this context, the overlap between regions with water stress and a good solar resource makes solar desalination an attractive technology option,^11^ as shown in Figure 1. For example, sun-rich and water-scarce regions such as Spain, Australia, and the southwestern United States are developing solar desalination systems, while hydrocarbon-rich nations in the Middle East are adopting solar energy to limit the carbon footprint of desalination.^14^

Despite this potential, renewable desalination as a whole accounts for only 1% of the global installed capacity.^15^ This can be attributed to the higher current cost of solar energy generation, resource intermittency, and the higher desalination capital cost at smaller scales. However, these trends are expected to change with the global transition to a decarbonized electric grid resulting in a rapid decrease in the cost of solar technologies—for example, the U.S. Energy Information Administration estimates that electricity from photovoltaics (PV) is now competitive with new natural gas combined cycle power plants and will be even cheaper by 2030,^16^ while solar-thermal (ST) process heat has the potential to replace natural gas-based heating.^17^ Resource intermittency has to be addressed with some form of *energy storage*,^18^ but storage needs for desalination have received little attention as existing water treatment plants are predominantly fossil fuel driven. Without energy storage, utilization of the capital-intensive equipment will be low which contributes to a high levelized cost, while such intermittent operation would also cause technical challenges as we discuss later.

**Figure 2:**
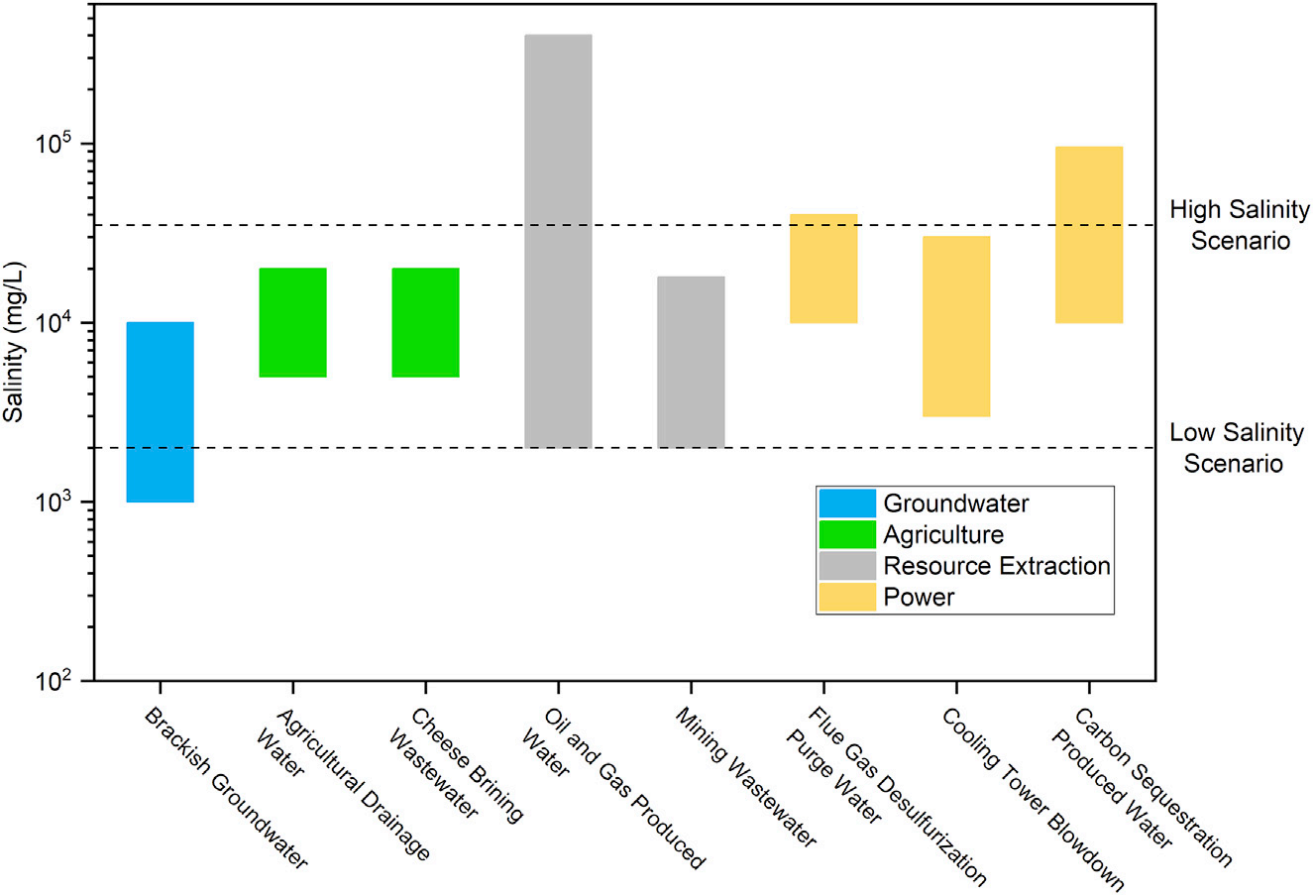
Salinity ranges of nontraditional water sources for distributed desalination Nontraditional water sources are grouped into different sectors—brackish groundwater and agriculture,^22^ resource extraction,^23^^,^^24^ and power generation.^25^^,^^26^ The broad salinity range is represented in terms of two scenarios for desalination: low salinity (2,000 mg/L) and high salinity (35,000 mg/L).

Another trend that is expected to favor solar desalination is the shift toward *decentralized or distributed desalination*.^7^ Conventional seawater desalination benefits from economies of scale, which has led to the establishment of large treatment facilities (capacities of over 50,000 m^3^/day) along the coastline accompanied with massive distribution systems that transport seawater to the centralized plant and deliver product water to end users.^7^ Recent estimates suggest that water conveyance costs can even exceed treatment costs—for example, the electricity cost for pumping alone can account for up to 40% of the desalinated water cost in water-stressed regions like Central Asia.^19^ Concomitantly, with close to 60% of the global population located away from coastal regions, beneficial reuse of nontraditional water sources (*e*.*g*., brackish groundwater, agricultural drainage, and industrial discharges) is increasingly of interest.^7^^,^^20^^,^^21^ Furthermore, as the energy sector continues to decarbonize, there will be additional nontraditional sources, including wastewater from battery materials mining and carbon sequestration. These are in predominantly inland locations, have a smaller volume (∼1,000 m^3^/day) due to their distributed nature, and have a range of salinities as shown in Figure 2.^8^^,^^23^^,^^25^^,^^27^^,^^28^

The emergence of distributed desalination also brings an often-overlooked component to the forefront—the cost of brine (a byproduct of desalination) management and/or disposal. A recent study showed that global brine production exceeds clean water production by about 50% owing to low water recoveries.^8^ While this is not a challenge for seawater facilities that discharge brine into the ocean, inland locations face restrictions with surface discharge due to the environmental impact of hypersaline brine (>50,000 mg/L) while deep-well injection has geographic limitations as well as economic and environmental costs.^6^^,^^29^ There is thus a strong driver to pursue zero liquid discharge (ZLD) or minimal liquid discharge technologies as a *brine management* strategy for desalination of these nontraditional sources.^30^^,^^31^ Furthermore, ZLD can have other positive impacts, such as resource recovery and/or valorizing the solids produced.^32^

The foregoing trends set a new paradigm for solar-driven desalination system at a distributed scale of ∼1000 m^3^/day with storage and brine management, which is markedly different from centralized desalination of seawater.^19^^,^^33^^,^^34^ This requires a new system design and technoeconomic framework that is not covered in the existing literature—for example, although there are comprehensive reviews on the integration of solar energy with seawater desalination,^35^ these analyses use grid back-up^36^^,^^37^^,^^38^ or cogeneration schemes^39^^,^^40^^,^^41^^,^^42^ to minimize intermittent operation and evaluate large-scale systems with treatment capacities >10,000 m^3^/day. On the other hand, there are reports on small-scale solar evaporation-based desalination with^43^ and without^44^^,^^45^^,^^46^^,^^47^ energy storage, but these are at capacities <1 m^3^/day. Furthermore, there is little literature on solar-driven ZLD—a recent analysis by Panagopoulos showed that brine treatment has the potential to be economically viable compared to brine disposal, but this was for a small-scale system of <50 m^3^/day.^48^ There is thus the need to establish the potential of a *holistic* distributed desalination system with storage and brine management.

In this perspective, we evaluate the levelized cost of water (LCOW) for various solar desalination systems (∼1000 m^3^/day capacity) comprising different combinations of energy source (electricity and heat), storage (battery, thermal storage, and water storage), desalination plant (membrane and thermal), and brine management (disposal or ZLD). These systems are benchmarked against conventional fossil fuel-driven desalination, and cost projections are made for 2030 based on renewable energy generation/storage targets set by the U.S. Department of Energy. The framework is then utilized to answer the following important questions for distributed desalination: *(i)* how does the integration of energy storage to address solar intermittency affect the water cost? *(ii)* what is the economic viability of adopting ZLD as a brine management strategy for inland facilities? *(iii)* what are the research gaps that can drive down the future cost of solar desalination to achieve parity with fossil fuels?

### Technology options and system design

A holistic system design for solar desalination (with a daily freshwater production capacity of 1000 m^3^) comprises four main subsystems: energy generation/source, desalination plant, storage unit, and brine management. To account for the broad range of nontraditional water salinities^20^ shown in Figure 2, two different water source scenarios are considered: 2,000 mg/L (low-salinity) and 35,000 mg/L (high-salinity). These values also correspond to commercially available desalination technology options for brackish groundwater and seawater, respectively, thus allowing for reasonable cost estimates from mature technologies. In addition, it is assumed that the desalination plant and the brine concentration subsystem (when present) rely on distributed solar energy resources that are self-sufficient (i.e., without grid backup) under a direct normal irradiance (DNI) of 6 kWh/m^2^ (typical value for water-stressed regions, as shown in Figure 1).^49^ As a result, no net power exchanges with the grid are required,^50^ unlike previous analyses in the literature.

For the energy source, three different technologies can be used: PV electricity, solar-thermal electricity (STE, with optical concentrators to produce high-temperature heat ∼400°C that is converted into electricity with a turbine, *i*.*e*., concentrated solar power), and solar-thermal heat (STH, with non-tracking collectors to achieve temperatures ∼150°C that are used as heat). An in-depth review of the different solar generation technologies can be found elsewhere.^35^ For the desalination plant, two processes are considered that comprise over 90% of installed capacity:^8^ membrane-based reverse osmosis (RO) driven by electricity and multi-effect distillation (MED) driven primarily with heat and a small electricity input. Intermittent operation of these processes has been shown to exacerbate membrane degradation and/or heat exchanger scaling and can also lead to complex ramp up/ramp down procedures.^51^^,^^52^ We thus argue that the desalination plant should run continuously, *i*.*e*., at a capacity factor (*CF*) close to unity. This is achieved using a storage subsystem comprising either battery energy storage (BES) or thermal energy storage (TES) to minimize fluctuations in solar energy. TES is further classified into low-temperature storage (LTTES—*e*.*g*., hot water or pressurized hot water) used directly as heat for STH and high-temperature storage (HTTES—*e*.*g*., molten salts) for conversion to electricity in STE.

**Figure 3:**
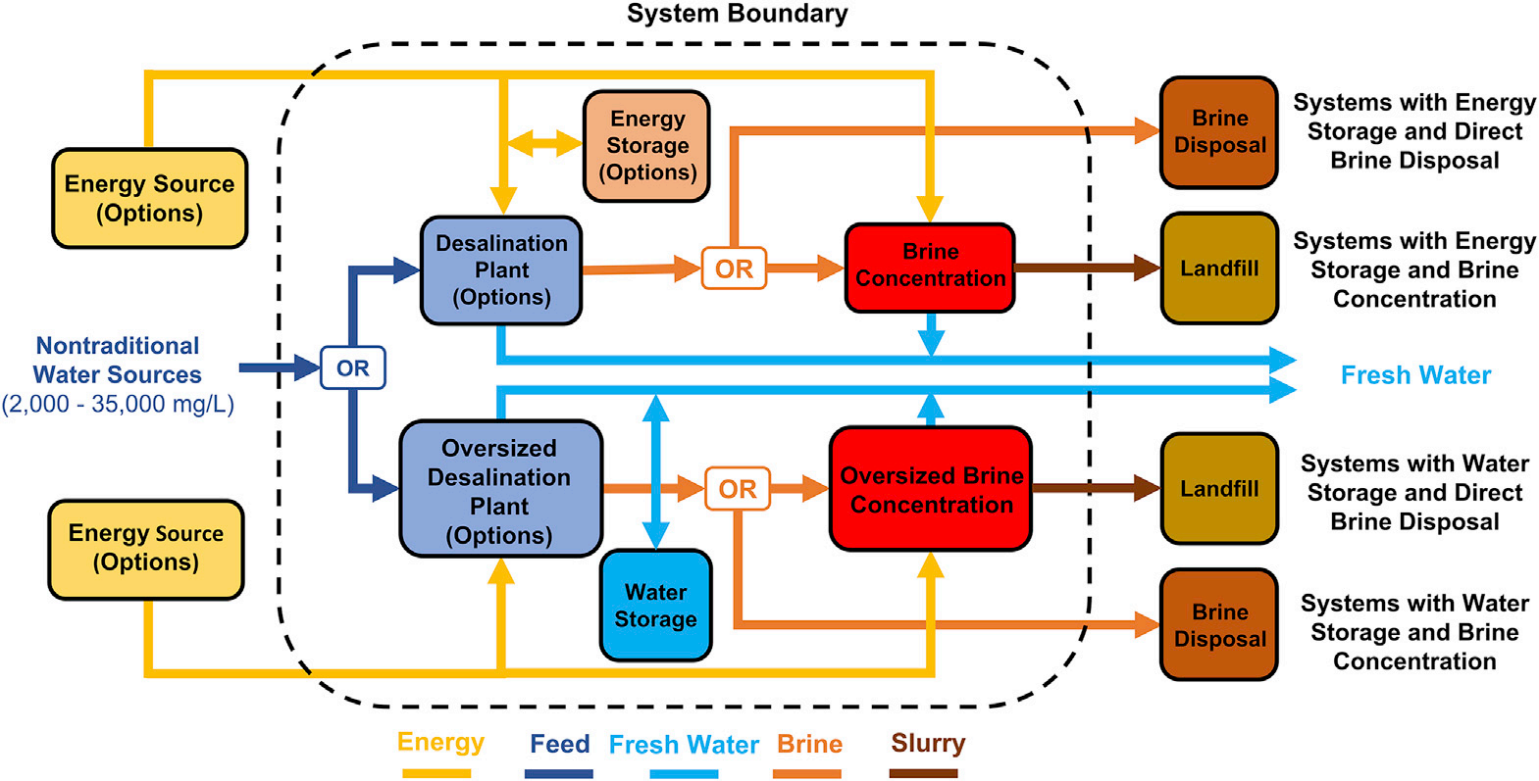
Schematic overview of distributed solar desalination systems Each system comprises choices about the energy generation, desalination plant, energy or water storage, and brine management (brine disposal or ZLD). The lower half of the schematic corresponds to systems with water storage, which are operated intermittently (*CF* = 0.25) with an oversized desalination plant and brine concentration unit. The system boundary is shown by the dashed line; the energy source is represented as a levelized energy cost (electricity or heat); the brine disposal process is treated as an additional cost per unit volume of brine disposed, while all other subsystems are explicitly modeled by their capital costs in this analysis. Figure S1 shows the 16 different system configurations, all of which are special cases of this overview figure.

An alternative to energy storage is water storage (WS) in a large tank. In this case, however, the desalination plant must be oversized to produce water while operating only during solar hours, *i*.*e*., *CF* = 0.25. WS not only results in technical limitations with intermittent desalination plant operation but also does not offer any economic advantage over energy storage as we show later. Finally, since the desalination plant has a limited recovery ratio (RR) (set by pressure limits of polymer membranes in RO and boiling point elevation in MED),^8^^,^^53^ the resulting brine must be further treated or disposed. Here, we consider two options for inland facilities: brine disposal by deep-well injection (DWI) in underground reservoirs (other disposal options are not suitable for hypersaline water)^54^^,^^55^ and brine concentration to ZLD via mechanical vapor compression (MVC) using electricity (state-of-the-art brine concentrator commonly used in ZLD schemes).^30^^,^^46^^,^^56^^,^^57^^,^^58^^,^^59^^,^^60^ Overall, these different energy source-storage-desalination-brine management options result in 16 system configurations, which are divided into four categories as shown in Figure 3: systems with energy storage and brine disposal, energy storage and brine concentration, water storage and brine disposal, and water storage and brine concentration.

For brevity, only 8 representative configurations that correspond to the most cost-competitive options are dicussed in the main text as shown in Table 1. Additional details of all other system configurations considered in this study are in the Methods section and in Table S1 and Figure S1 of the Supplemental Information section. The subsystem power consumptions and the subsystem sizes are also presented in Tables S7 and S8, respectively.

**Table 1:** Down-selected system configurations for distributed solar desalination with storage and brine management^a^^,^^b^^,^^c^

Desalination System with:	Configuration Name *(and Config. # in the SI)*	Electricity Source	Thermal Energy Source	Electricity Storage	Thermal Storage	Desalination Plant	Brine Management
Energy Storage and Brine Disposal ***(45% recovery)***	PV – RO with BES and DWI *(Config. 1)*	PV	–	BES	–	RO	DWI (brine disposal)
	STH – MED with LTTES and DWI *(Config 3a)*	STE	STH	HTTES	LTTES	MED	
	STE – RO with HTTES and DWI *(Config. 4)*	STE	–	HTTES	–	RO	
Water Storage and Brine Disposal ***(45% recovery)***	PV – RO with WS and DWI *(Config. 5)*	PV	–	–	–	RO (4x oversized)	
Energy Storage and Brine Concentration ***(95% recovery)***	PV – RO with BES and MVC *(Config. 9)*	PV	–	BES	–	RO	MVC (zero-liquid discharge)
	STH – MED with LTTES and MVC *(Config. 11a)*	STE	STH	HTTES	LTTES	MED	
	STE – RO with HTTES and MVC *(Config. 12)*	STE	–	HTTES	–	RO	
Water Storage and Brine Concentration ***(95% recovery)***	PV – RO with WS and MVC *(Config. 13)*	PV	–	–	–	RO (4x oversized)	MVC (4x oversized)

PV, photovoltaics; STE, solar-thermal electricity; STH, solar-thermal heat; BES, battery energy storage; TES, thermal energy storage; HTTES, high-temperature TES; LTTES, low-temperature TES; WS, water storage; RO, reverse osmosis; MED, multi-effect distillation; MVC, mechanical vapor compressor; DWI, deep-well injection.

a See Table S1 and in Figure S1 for the full set of 16 configurations analyzed.

b This represents the high-salinity scenario, while the low-salinity scenario does not include any thermal desalination configurations, as described later (also see Table S9).

c Configurations with energy storage operate at a capacity factor of 1. Water storage results in a capacity factor of 0.25, owing to which the desalination plant (and brine concentration unit, when present) is oversized.

To benchmark these solar desalination systems against state-of-the-art desalination powered by fossil fuels, 4 baseline system configurations are also analyzed (see Table S2 and Figure S1). A key difference is that energy or water storage is not required in these baseline cases, and the configurations include combined cycle gas turbine electricity (CCGTE)-RO with DWI brine disposal, natural gas heat (NGH)-MED also with DWI, CCGTE-RO with MVC to achieve ZLD, and NGH-MED also with MVC. Electricity demands of both MED and MVC are fulfilled by CCGTE.

### Technoeconomic modeling framework—LCOW

To compare the system configurations shown in Table 1 for distributed solar desalination, we present a levelized cost framework that accounts for capital costs, energy (or fuel) costs, fixed and variable operations and maintenance costs, financing costs, and utilization rates or *CF*s. Specifically, we introduce a comprehensive LCOW metric that includes storage and brine management/ZLD costs, which were not captured in previous technoeconomic analyses:^41^^,^^61^^,^^62^^,^^63^^,^^64^

(Equation 1) $$LCOW=\begin{array}{c} \sum_{i}\frac{{CAPEX}_{i}\times {Size}_{i}\times \frac{r{\left(1+r\right)}^{ni}}{{\left(1+r\right)}^{ni}-1}}{CF\times \left({Size}_{desal}+{Size}_{ZLD}\right)\times 365}+{OPEX}_{fix}+{OPEX}_{DWI}+{OPEX}_{var}\\ i=desalination\;plant,storage\;\left(battery,thermal,or\;water\;storage\right),ZLD\;unit\;\left(when\;present\right) \end{array}$$

Each term in Equation 1 and the associated input assumptions are described in detail in the Methods section, in Note S3, and the input values (with units) are shown in Table S3. Briefly, in the first term of Equation 1, the numerator is the amortized installed capital expenditures (CAPEX) of the desalination plant, storage unit, and ZLD subsystem (when present). The CAPEX ($/(m^3^/day)) is amortized over the entire 30-year system lifetime for all subsystems except batteries, for which CAPEX is amortized over a 10-year lifespan (*n*_*desal*_*, n*_*TES*_*, n*_*WS,*_ *n*_*ZLD*_ = 30 years, *n*_*BES*_ = 10 years). This amortized cost is divided by the total freshwater production in a year from the desalination plant and ZLD unit (when present). The OPEX_fix_ term ($/m^3^) represents the fixed operations and maintenance expenditures, OPEX_repl_ ($/m^3^) accounts for the replacement costs of components that have a shorter lifetime than the system lifetime, and OPEX_DWI_ ($/m^3^) represents the cost of brine disposal through deep-well injection for system configurations without ZLD.^55^ The final term, OPEX_var_ ($/m^3^), is the variable operational cost, which is dominated by the cost of energy required for the desalination unit and ZLD unit (when present). This is expressed as the product of the levelized cost of energy and the specific energy consumption (SEC) of desalination and ZLD (when present). For PV and STE, the energy cost is given by the levelized cost of electricity (LCOE), while we calculate the cost of thermal energy associated with STH as the levelized cost of heat (LCOH) as shown in the Methods section. Overall, the solar energy generation subsystem is represented in terms of a levelized energy cost (as electricity or heat), and the brine disposal subsystem is treated as an additional cost per unit volume of brine disposed, while all other subsystems (desalination, storage, and ZLD) are explicitly modeled using their capital costs (see Note S3).

## Results and discussion

Using the framework described above and the input assumptions given in Table S3, the LCOW values for different solar desalination system configurations are calculated. We find the overall trends are similar for the low- and high-salinity scenarios (see Figures S2 and S5), and thus we focus on the high-salinity results for brevity, followed by a brief discussion on low salinity.

### Fossil fuel-based desalination—Technology baselines

**Figure 4:**
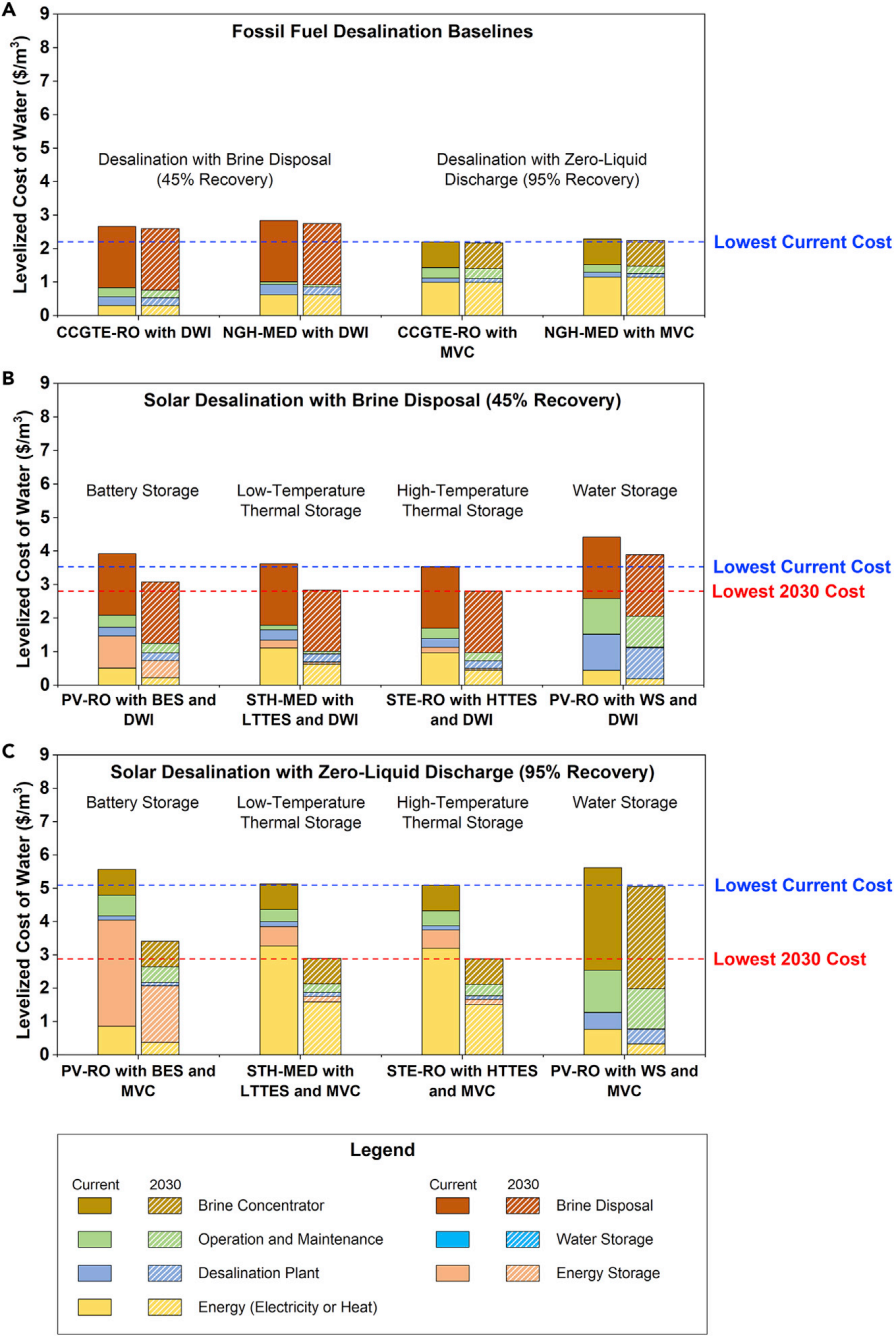
LCOW for different distributed desalination system configurations with brine management showing the current cost breakdown (solid bars) and 2030-projected costs (dashed bars) with a desalination input salinity of ∼35,000 mg/L (A) Fossil fuel (natural gas)-driven desalination baseline systems with 45% water recovery followed by DWI brine disposal and 95% water recovery using MVC. (B) Solar desalination systems with energy and/or water storage achieving 45% water recovery followed by DWI brine disposal. (C) Solar desalination systems with energy and/or water storage achieving 95% water recovery using an MVC brine concentrator. All configurations and baselines are specified in Note S1.

The LCOW of fossil fuel-based desalination baselines is shown in Figure 4A. We note that the exact LCOW depends on financing and energy costs, leading to variations of ∼25% or more based on the region. Here we use values typically seen in the United States, but the trends and conclusions can be extended to other parts of the world using this technoeconomic framework. For the CCGTE-RO and NGH-MED baselines with 45% water recovery, when we ignore the DWI brine disposal costs (dark brown component of the bars), the LCOW approaches $1/m^3^ which is consistent with seawater desalination followed by ocean discharge.^65^^,^^66^ However, when the brine disposal cost is included, the LCOW increases by a factor of 3×. This significant cost increase reveals the economic impact of desalination brine, making it a major challenge for inland treatment facilities. This is further complicated by the fact that DWI is permitted only in certain locations and has an adverse environmental impact that is not quantified in this analysis. These limitations motivate the use of brine concentration technologies that increase water recovery to 95% (ZLD). Now comparing the CCGTE-RO and NGH-MED baselines with an MVC brine concentrator for achieving ZLD in Figure 4A, we find that the cost of water produced is double (∼$2/m^3^) that of conventional seawater desalination with ocean discharge. However, when the DWI disposal cost is included, the two baselines with MVC have a lower LCOW. This suggests that for fossil fuel-driven inland desalination systems, the benefit of not having to dispose a large volume of brine through DWI outweighs the MVC capital and electricity costs for achieving ZLD. This is the first quantification of the economic advantage of ZLD over brine disposal for distributed desalination, and it underscores the feasibility of transitioning to a circular water economy. We also note that DWI costs can vary from $0.33–2.64/m^3^ (the chosen value of $1.5/m^3^ is the average) owing to differences in the depth and diameter of the well for different volumes.^54^^,^^55^ Reducing the DWI cost to $0.5/m^3^ changes the conclusion about ZLD being more cost-effective than brine disposal (see Note S7 and Figure S4). However, cost is not the only consideration for DWI as it has adverse environmental impacts, in addition to geologic and regulatory restrictions that serve as drivers to pursue ZLD in inland locations.^30^

### Solar desalination with brine disposal (45% water recovery)

The LCOW for solar desalination with 45% water recovery followed by DWI brine disposal is shown in Figure 4B. When PV-generated electricity is used to drive an RO plant (PV-RO with BES and DWI), the LCOW is higher than the corresponding baseline (CCGTE-RO with DWI), primarily due to the high CAPEX of battery storage for continuous operation. Even if the LCOE for utility-scale rather than commercial-scale PV is used—which is currently competitive with natural gas prices—the LCOW remains nearly unchanged. Alternately, STH can be used to drive an MED plant with LTTES (STH-MED with LTTES and DWI). The relatively small electricity consumption of MED can be supplied either by PV with BES or by STE with HTTES, with the latter being the cheaper option (see Note S4). For this configuration then, the LCOW is higher than that of the corresponding baseline (NGH-MED with DWI) owing to the higher current cost of solar heat. However, this LCOW is lower than the PV-RO configuration, due to the low cost of thermal energy storage. A third option in Figure 4B is to use STE with HTTES to power an RO plant (STE-RO with HTTES and DWI) which yields the lowest LCOW of 3.5 $/m^3^ by combining inexpensive thermal storage with energy-efficient membrane desalination. A comparison of these three systems reveals that despite the low cost of PV, solar-thermal generation (either as STE or STH) is the more economical energy source for desalination.

The integration of energy storage allows these configurations to run around the clock, *i.e., CF* = 1. Alternatively, the desalination plant can be operated only during solar hours (*CF* = 0.25), with water storage being used to meet the daily production capacity (PV-RO with WS and DWI in Figure 4B). However, desalination with energy storage outperforms systems with water storage from a cost standpoint, as shown in Figure 4B. For example, in PV-RO with water storage, the high cost of battery storage is avoided, but this is offset by the higher CAPEX of an oversized desalination plant with low utilization. Furthermore, as previously discussed, desalination plants are designed to operate continuously, making water storage also impractical from a technology standpoint even though the storage tank cost itself is negligible. A similar trend has been reported in the literature,^36^ which suggests that investments in energy storage for desalination are necessary.

Recently, there has been a push toward widespread electrification to achieve a renewable grid at a low cost. In this case, PV electricity can be used for resistive heating with LTTES to drive MED desalination. However, this configuration has the highest cost among the 16 systems analyzed due to the high thermal energy consumption of MED that is produced from renewable electricity and thus is omitted from Figure 4B (see Note S4). Using this framework, we find that electrification of heat for desalination would only be favorable if the LCOE of PV becomes lower than $0.01/kWh_e_. In fact, even if resistive heating is replaced with a heat pump that has a coefficient of performance (COP) of 3, the LCOW of PV-driven MED reduces to 4.3 $/m^3^, which is still higher than many of the other configurations. However, it is important to note that PV-driven industrial heat pumps will be an important component for providing efficient and emission-free heat for emerging thermal desalination processes (*e.g.,* membrane distillation, humidification-dehumidification, etc.).^67^^,^^68^

### Solar desalination with brine concentration to ZLD (95% water recovery)

The solar desalination systems discussed thus far have limited water recovery and generate brine that requires disposal. To concentrate the brine to ZLD, the same energy generation-storage-desalination units can be used, but each system now includes an MVC unit (powered by either PV or STE during daytime and by either BES or HTTES during nighttime) instead of brine disposal by DWI. In these cases, 95% water recovery is achieved and the remaining slurry (high solids content) is disposed in a landfill at a negligible cost. As shown in Figure 4C, the solar-driven ZLD is currently 1.5× more expensive than the corresponding brine disposal configurations of Figure 4B. In contrast, Figure 4A shows that for fossil fuel-driven desalination, ZLD is actually slightly cheaper than brine disposal. For PV-RO with BES and MVC, the higher LCOW is dominated by the prohibitively high cost of battery storage to power both the RO plant and the MVC unit during hours of low/no solar insolation. In comparison, solar-thermal configurations with thermal storage (STH-MED with LTTES and MVC and STE-RO with HTTES and MVC) have lower costs, but the LCOW is still high when compared with brine disposal configurations owing to the cost of STE to drive MVC. For solar-ZLD to become competitive with its fossil fuel counterpart, our analysis suggests that the MVC energy consumption and/or energy cost (electricity) should be significantly reduced, as we discuss in the Outlook section. Again, for systems with ZLD, water storage for ZLD offers no economic advantage over energy storage.

### LCOW projections to 2030 with reduced energy and storage costs

The analysis so far revealed that the two main factors that contribute to a higher current LCOW for solar desalination compared to conventional fossil-driven desalination are the costs associated with *(i)* generating solar energy and the *(ii)* storage of this energy to address intermittency. These two factors are not unique to desalination and are also the focus of considerable R&D for a renewable electric grid. SunShot targets set by the U.S. Department of Energy include a foreseeable decrease in the cost of solar harvesting technologies (both PV and STE/STH) and energy storage technologies (both BES and TES) by 2030. Incorporating these targets in the techno-economic framework, along with desalination annual learning rates (13% and 23% for RO and MED, respectively, to model reduction in plant capital costs), the projected LCOW by 2030 is calculated (see Table S4).^39^^,^^40^ Note that these learning rates are for water treatment plants with capacities of ∼1000 m^3^/day corresponding to distributed desalination, rather than based on economies of scale. As shown in Figures 4B and 4C (dashed bars), all configurations with energy storage are expected to see a 20–50% decrease in the overall LCOW by 2030 due to projected reductions in LCOE and LCOH, as well as storage and desalination capital costs. In addition, for both brine disposal and ZLD systems, solar thermal desalination with thermal storage will continue to be the most economical options. Further details for the 2030 LCOW projections are provided in Note S5, and a discussion on emerging desalination processes beyond RO and MED by 2030 is included in the Limitations of the Study section.

### Sensitivity analysis

**Figure 5:**
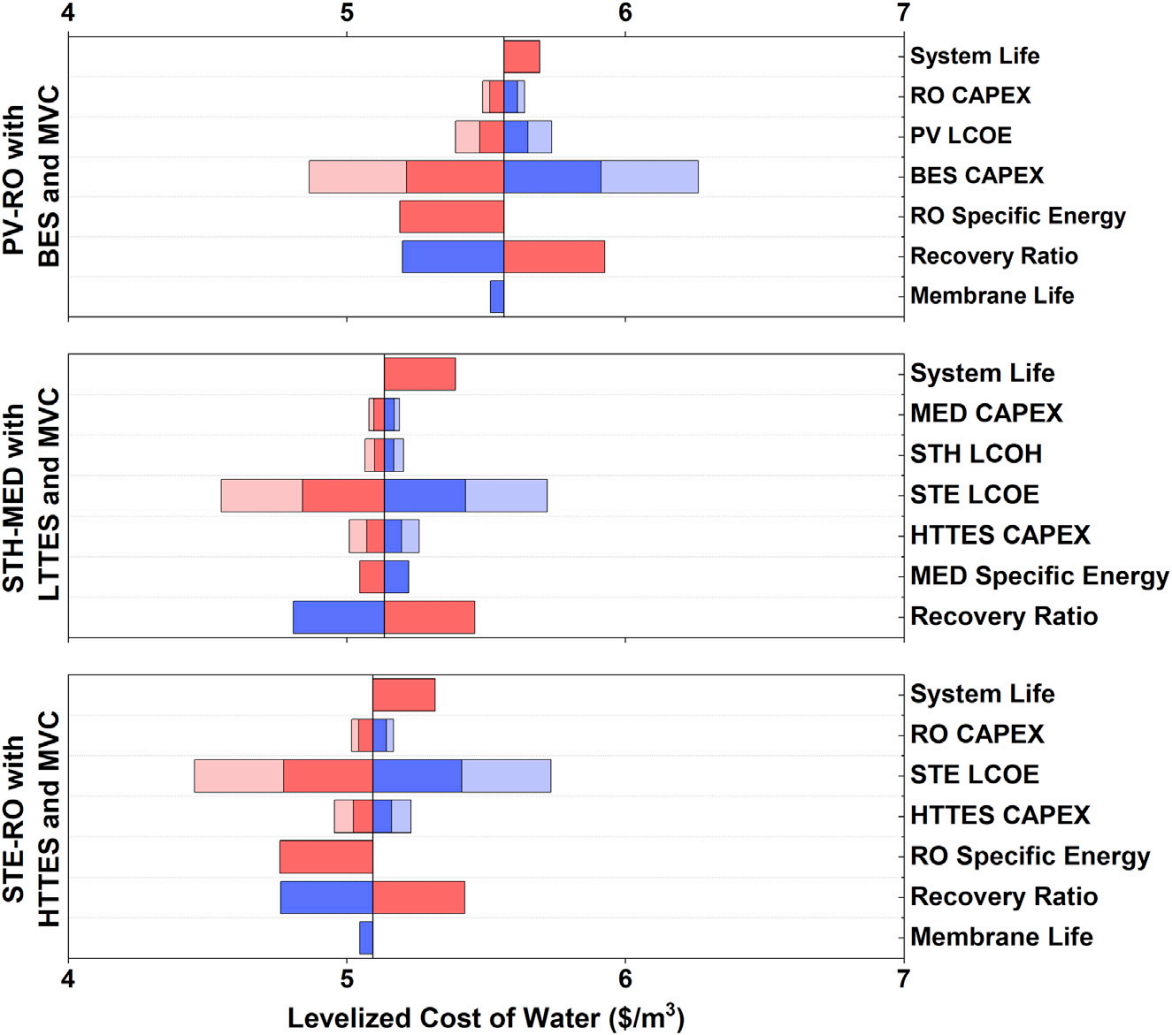
Single parameter sensitivity analysis for solar desalination systems that achieve ZLD and utilize energy storage for continuous operation (high-salinity scenario: ∼35,000 mg/L) Each calculation represents the variation of a single input parameter from the base value to the higher and lower values (in blue and red bars, respectively) that are specified in Table S5. For certain parameters such as BES CAPEX, two different ranges were evaluated, denoted by darker and lighter tints. For some parameters such as RO specific energy, the varied range is not symmetric as explained in Note S6.

To understand the impact of different input variables on the LCOW, a sensitivity analysis is also performed (see Table S5 for parameter ranges). Only configurations using energy storage and MVC brine concentration are analyzed (specifically, the first three scenarios of Figure 4C) as they are most suitable for inland and/or distributed desalination. For PV-RO with BES and MVC, the LCOW is most sensitive to the capital cost of batteries to store electricity for the desalination and ZLD subsystems. The water recovery ratio and RO specific energy also play important roles, as shown in Figure 5. In comparison, for systems that use thermal storage, LCOW is most strongly influenced by the cost of STE required primarily to run the MVC unit, while longer system lifetimes and higher recovery ratios lower the LCOW. The latter is because a higher desalination recovery ratio reduces the volume of brine that needs to be treated, thereby requiring a smaller MVC unit to meet the total daily water demand of 1000 m^3^/day. Interestingly, LCOW is insensitive to the RO capital cost and membrane lifetime, indicating that these parameters are not worthwhile to optimize further in a ZLD system from a cost perspective. Similarly, the MED capital cost and thermal storage cost are relatively unimportant for LCOW. Notably, the sensitivity analysis confirms that the major trends remain unchanged after accounting for large uncertainties in input parameter costs. In other words, solar-thermal-driven desalination coupled with either RO or MED and thermal energy storage is the optimal system design.

### LCOW for the low-salinity scenario

**Figure 6:**
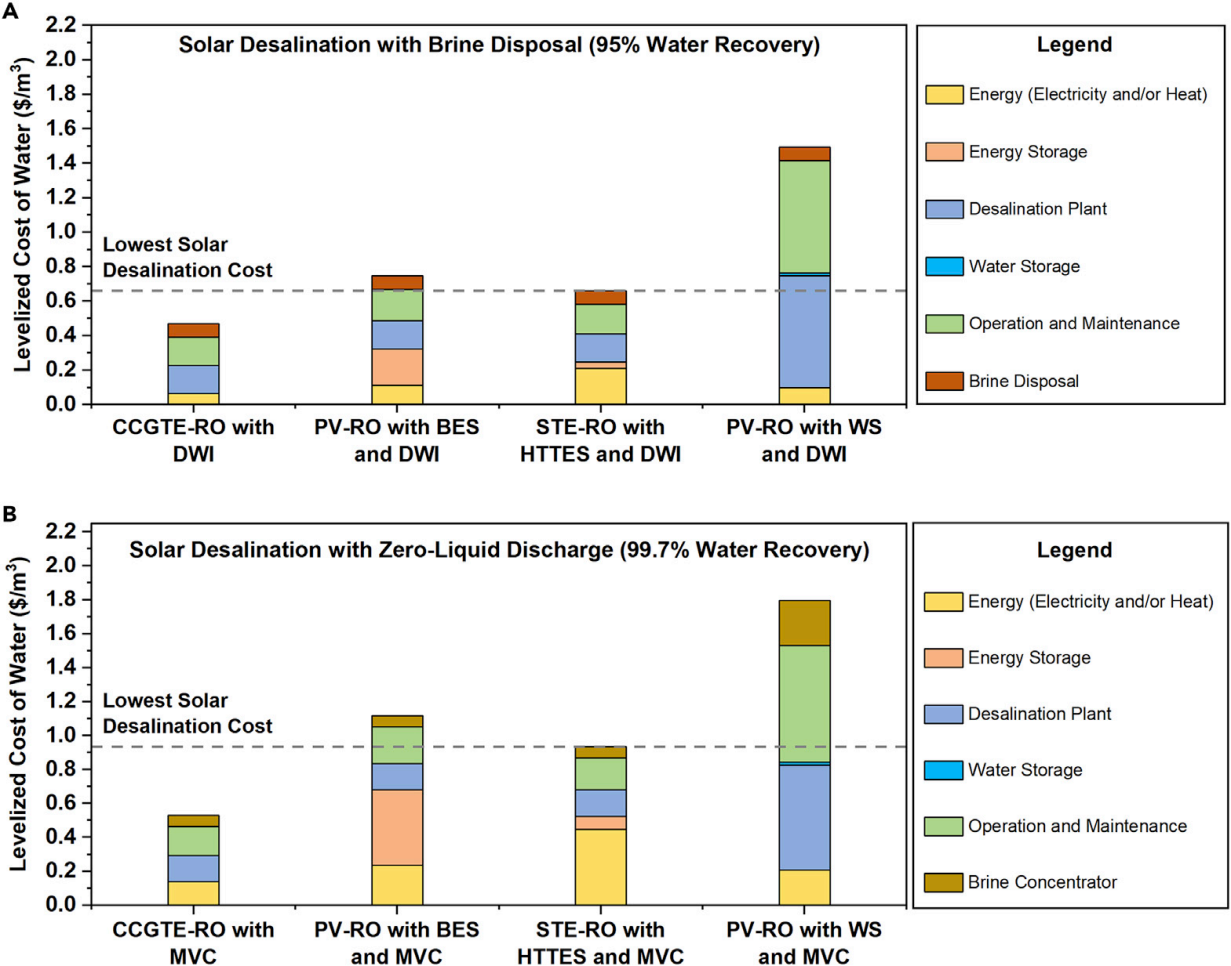
LCOW for distributed desalination systems at low salinity (2,000 mg/L) (A) Desalination with 97% water recovery from RO followed by DWI brine disposal and (B) Desalination with brine concentration to ZLD achieving 99.7% water recovery. The configurations are specified in Table 1, the power consumptions are in Table S7, the subsystem sizes are in Table S8, and the input parameters are shown in Table S9.

This techno-economic analysis framework can also be applied to identify the cost-optimal system design for nontraditional water sources with a low salinity ∼2,000 mg/L. Under these conditions, thermal desalination using MED is known to be inefficient,^60^ resulting in only RO-based systems being analyzed (power consumption and subsystem sizes are shown in Tables S7 and S8, respectively). The major differences are the lower desalination capital cost, specific energy consumption, and higher water recovery for the low-salinity scenario (see Table S9); the RO brine at 60,000 mg/L goes into the same MVC unit as the high-salinity case. All other inputs to the techno-economic model are assumed to be the same as the high-salinity scenario. As expected, the LCOW is significantly lower (by a factor of 3-4×) compared to the corresponding high-salinity scenario, with the fossil fuel baseline cost being in same range as the literature values for brackish water RO.^69^ This is shown in Figure 6, and the overall trends are similar to the high-salinity case with STE-RO being slightly cheaper than PV-RO. The other main takeaway is that for the low-salinity scenario, energy storage is again superior to water storage, suggesting that energy storage is the best approach to address solar intermittency at all salinities.

## Outlook: Research and development directions

This comprehensive levelized cost analysis of solar desalination at different salinities representative of nontraditional water shows that technology innovation is necessary to achieve cost parity with conventional fossil fuel-driven desalination. To this end, we outline four research and development priorities and set cost targets for the energy and water subsystems.

### Modular STE systems

Solar-thermal desalination with thermal storage has the lowest system-level cost based on this analysis. However, this cost can be reduced further because the LCOE of Stirling dish systems used herein is ∼2× higher than state-of-the-art concentrated solar power plants (LCOE of power tower systems rated at >50 MW_e_ capacity with 12 h of thermal storage was $0.098/kWh_e_ in 2018).^70^ There is thus an opportunity to develop low-cost, modular solar-thermal systems at capacities <5 MW_e_ with storage to enable distributed solar desalination. Energy storage options include thermal (latent heat) or chemical (hydrides), but their performance and durability are yet to be demonstrated at high temperatures.^71^ Tied to this is the need for turbines with conversion efficiencies >30% at small scales where steam turbines are inefficient (∼100 kW_e_–1 MW_e_). The development of next-generation turbines, such as supercritical CO_2_ turbines or micro-turbines that run on the Brayton cycle, may be important to realize modular STE. Specifically, the cost of modular STE technologies with integrated thermal energy storage should be reduced to <$0.06/kWh_e_ to realize the full potential of this technology. For example, at an LCOE of 0.05 $/kWh_e_, solar-driven ZLD will not only be more economically viable than brine disposal but will also approach cost parity with fossil fuel-driven ZLD.

### Low-cost electricity storage

PV electricity is competitive with industrial natural gas prices in the U.S. on a levelized cost basis, but this is outweighed by the need for battery storage to minimize intermittent operation of the desalination plant. Over 90% of large-scale battery storage was provided by Li-ion batteries in 2019,^72^ which results in a high LCOW for the desalination system even with projected cost reductions by 2030. This underscores the need for low-cost and long-duration (>10 h) electricity storage technologies—for example, a total installed storage cost of 40–70 $/kWh_e_ suggested by Albertus et al. would make PV-RO the most economic desalination system configuration.^73^ This may be achieved with flow batteries,^74^^,^^75^ but the integration of such an electricity storage subsystem with PV-RO requires further investigation. Extending this to ZLD systems, with a battery storage cost of $50/kWh_e_, PV-RO with BES and MVC will be more economically viable to its brine disposal counterpart, and this configuration will close in on the gap to achieve cost parity with fossil fuel-driven ZLD.

### Adaptive and process-intensified desalination

Given the dominance of energy storage cost on the LCOW, new desalination processes that can adapt to the intermittency of renewable sources (*e.g.,* variable flowrate or partial load operation) can yield entirely different cost-performance trends not captured in this analysis.^76^ This is especially important given the limitations of RO and MED under intermittent operation that results in severe reduction in permeability and fluctuations in distillate purity, respectively.^77^^,^^78^ Thus, research efforts on developing adaptive as well as process-intensified desalination technologies^7^ that achieve higher water recoveries (>50% for a salinity of ∼35,000 mg/L) can have a significant impact on the water cost by reducing the brine volume that needs treatment and/or disposal. For example, high-recovery RO configurations (*i.e.,* high-pressure RO, osmotically assisted RO, cascading osmotically mediated RO, etc.)^79^^,^^80^^,^^81^^,^^82^ are promising but still in very early stages of development.

### Thermal brine concentrators

Finally, given the dominance of MVC capital and energy costs in ZLD systems, development of new brine concentrators is necessary. The energy cost of MVC is tied to the cost of electricity, and even with projected LCOE reductions by 2030, solar-driven ZLD will not be cheaper than fossil fuel ZLD with its LCOW of only ∼$2.2/m^3^. Compared to electricity, solar heat is an order of magnitude cheaper (see Table S6), which indicates an opportunity to develop thermally driven brine concentrators that can be coupled with a low-cost TES. Emerging treatment technologies are promising in this regard as they comprise inexpensive materials and can be driven by heat (*e.g.,* membrane distillation and humidification-dehumidification)^83^ and in some cases leverage non-evaporative thermal phase transitions to be more energy-efficient (*e.g.,* forward osmosis and solvent extraction).^45^^,^^84^^,^^85^ Pilot-scale demonstrations using salinities higher than seawater^86^ will be needed to assess viability under scale-inducing conditions in ZLD.

## Limitations of the study

Given that the primary goal of this work is to analyze the potential for solar desalination with energy storage (fully decarbonized water treatment system) and to evaluate the impact of brine management for distributed inland desalination, the scope is limited to LCOW analysis of such scenarios. Furthermore, we highlight that the techno-economic framework developed herein can be modified and/or extended to analyze other desalination technologies provided that capital and operating (primarily energy) costs are available. Below, we outline some of the limitations of the study, and all assumptions are detailed in Note S2 and Table S3.

Scope: The decarbonized configurations analyzed in this work are driven solely by solar energy (as electricity or heat or a combination of both), and no hybrid systems (*e.g.,* cogeneration-driven desalination or waste heat recovery) are considered although these may be more energy efficient. These systems are intentionally excluded from the scope of this work as their techno-economics have already been reported in the literature.^39^^,^^41^^,^^42^ Within solar desalination configurations, only mature technologies with well-reported cost and performance data at the ∼1000 m^3^/day scale have been modeled in this work. Recently, there has been work on assessing the cost and performance of solar-driven commercial-scale membrane distillation (MD), including air-gap, permeate-gap, and vacuum MD.^86^^,^^87^ The modular nature of this process makes it attractive for distributed desalination (prototypes up to 100 m^3^/day have been demonstrated), while the ability to operate at higher salinities under ambient pressure and intermittent conditions makes it promising for ZLD. The LCOH and SEC (which in turn depends on the gained output ratio or thermal efficiency) dominate the LCOW, with values ranging from $0.3–18/m^3^ depending on the heat source and feed salinity.^88^^,^^89^ Schwantes et al. performed a detailed cost comparison of MD and MVC for brine concentration; this study revealed that both the air-gap and vacuum MD configurations are more economical than MVC for ZLD at capacities between 10 and 1000 m^3^/day.^59^ However, the cost of steam is used as the heat source, and additional analysis on how integrating solar energy and storage would impact the water cost and long-term operation is needed.

Location-specific LCOW: To make the LCOW framework generalizable and with the focus primarily being on analyzing the effect of storage on water costs, no location-specific inputs (*e.g.,* land cost, water demand, population density, solar resource, transportation costs, etc.) have been considered. Specifically, to compare the different solar desalination configurations, we use an average DNI of 6 kWh/m^2^ for water-stressed regions with a good solar resource (see Figure 1) and a CF of 0.25.^90^^,^^91^^,^^92^ These inputs are used to size the desalination plant to produce 1000 m^3^/day of clean water with or without energy storage (24-h operation or only daytime operation, respectively). We note however that energy storage will also be needed to maintain near-constant power supply to both RO and MED (and MVC when present) for the WS configurations and address fluctuations in the solar flux. This additional energy storage (*e.g.,* batteries for RO and TES tanks for MED) is not included in the LCOW calculated herein. It is suggested that future studies incorporate these factors with the framework developed herein to obtain accurate LCOW estimates.

Impact of resource recovery on LCOW: While the LCOW equation presented as Equation 1 considers the impact of brine management on water costs, it does not account for resource recovery (i.e., transforming the waste brine into useful chemicals) or valorization of the solids (e.g., high-purity salts, magnesium, lithium, rare-earth elements, etc.) produced with ZLD. The economic value of these recovered products could offset the operational cost of ZLD processes, making this a promising avenue to lower the LCOW.^58^^,^^93^^,^^94^ However, this aspect of water treatment is in its infancy—with a wide variability in the possible resources that can be extracted, geographic considerations, and extraction techniques (membrane-based vs. adsorption, etc.), as well as the lack of literature on potential valorization costs—and is not considered in this study. Furthermore, ZLD may have unintended negative consequences wherein the solid waste produced is not suitable for reuse and can cause odors, harm wildlife, or even pose chemical leakage risks.^31^^,^^32^ This is turn may require disposal in hazardous waste facilities with an associated cost that is not considered in the present analysis.

Drawbacks of fossil fuel-driven desalination: The two main drawbacks of state-of-the-art desalination are the price volatility and CO_2_ emissions from fossil fuels (natural gas in this case). Carbon capture costs are not included in the analysis and would further increase the costs of the fossil fuel baselines calculated herein. For example, an estimated carbon capture cost^95^ of $0.058/kg CO_2_ would result in at least an additional ∼$0.3/m^3^ cost for the desalination baselines. Furthermore, an increase in the natural gas prices would impact some of the conclusions and comparisons with solar desalination: *(i)* if the natural gas LCOE doubles (compared to the 2020 value in Table S3), the baseline ZLD configurations (baseline 3 and 4) will be more expensive than brine disposal (baseline 1 and 2) and *(ii)* if the natural gas price triples (compared to the 2020 value in Table S3), the 2030 projected costs for solar desalination are comparable and even cheaper than fossil fuel desalination.

Cost projections: For the cost 2030 predictions, we primarily capture the effects of lower LCOE or LCOH and energy storage costs on the LCOW of existing desalination processes such as RO and MED (and MVC when present). However, by 2030, there may be transformative desalination technologies that have different costs and energy consumption that are not captured in the present analysis (*e.g.,* membrane distillation, solvent extraction, etc.), but the techno-economic framework developed herein can be applied once robust cost and performance data are available at a capacity of ∼1000 m^3^/day.

## Conclusions

Paradigm shifts in water treatment are underway to achieve a sustainable energy and water economy. This work develops a general system design and technoeconomic framework to evaluate the levelized water cost for distributed solar desalination (1000 m^3^/day) integrated with energy storage and brine management. The dominant factors that affect the overall LCOW are the costs of harvesting solar energy, storing that energy, and managing the brine in inland locations with limited disposal options. Investigation of different system configurations reveals that compared to PV, solar-thermal energy is economically favorable for coupling to desalination (both membrane and thermal) and zero-liquid discharge processes, owing to the lower cost of thermal storage than that of batteries. This underscores the benefit of a holistic analysis as the outcomes vary significantly from the literature on PV-RO with grid backup. The framework is also used to quantify the cost benefits of fossil fuel-driven ZLD over brine disposal for inland facilities for the first time. Furthermore, the analysis reveals that desalination with water storage is not viable despite the low cost of storage since this benefit is offset by the need for a larger desalination plant with a higher CAPEX. Finally, cost predictions to 2030 indicate that the levelized cost for distributed solar desalination will decrease significantly, approaching parity with conventional desalination. Overall, this work offers key insights and important techno-economic drivers for future R&D in renewable desalination.

## Methods

### System configurations

The different energy source-storage-desalination-brine management options yield 16 system configurations that are represented by the generic system topology of Figure 3 and are detailed in Note S1 (Table S1 and Figure S1). The first 8 configurations have a 45% desalination water recovery followed by brine disposal by DWI, and the other 8 configurations have a 95% water recovery by using a brine concentrator to achieve zero liquid discharge. For PV-RO with BES and DWI (also referred to as *Configuration 1* in Table 1 and in the Supplemental information), when solar energy is available during the daytime, PV-generated electricity is used to drive the RO plant and to simultaneously charge the BES subsystem. At night, the BES is discharged to power the desalination plant.

For STH-MED with LTTES and DWI (*Configuration 3a*), STH is used to drive an MED plant during daytime while charging the LTTES unit. STE is used to supply the smaller electricity load of the MED plant while also charging the high-temperature thermal storage unit. During nighttime, the LTTES subsystem drives the MED plant, while the HTTES output is converted into electricity to supply the MED plant.

For STE-RO with HTTES and DWI (*Configuration 4*), STE is used to power an RO plant and to charge the HTTES unit during daytime, whereas the high-temperature thermal energy stored in the HTTES is discharged and converted into electricity to power the RO plant during nighttime. Finally, in the PV-RO with WS and DWI configuration (*Configuration 5* in the Supplementary Materials), an oversized PV-RO desalination plant that operates at a CF of 0.25 is used to produce enough freshwater for the entire day when operating only during sunlight hours.^90^^,^^91^^,^^92^ The excess water produced during daytime is stored in WS for consumption during nighttime when the desalination subsystem is shut down. All of these 4 systems have limited water recovery and generate brine, which is then disposed by DWI at an associated cost (see Table S3 of the Supplemantary Materials). Note that DWI is geographically limited and has a significant environmental impact, which is not accounted for in the disposal cost.

Therefore, to find an alternative to direct brine disposal and to explore the techno-economic possibility of using MVC for achieving ZLD, 4 additional system configurations—PV-RO with BES and MVC (*Configuration 9*), STH-MED with LTTES and MVC (*Configuration 11a*), STE-RO with HTTES and MVC (*Configuration 12*), and PV-RO with WS and MVC (*Configuration 13*) are also included in the discussion (with results shown in Figure 4C). These 4 system configurations are designed with the same generation-storage-desalination subsystems as the other 4 configurations mentioned previously, but now instead of directly disposing the brine through DWI, an MVC unit (powered by either PV or STE during daytime and by either BES or HTTES with energy conversion during nighttime) is used to concentrate the produced brine into a slurry, which can be disposed in a landfill at a negligible cost.

### Levelized cost of water (LCOW)

Referring to Equation 1, CAPEX reflects the amortized capital investment, which includes the per unit capital cost of each individual subsystem (in $/(m^3^/day) for desalination and ZLD, $/kWh for energy storage, and $/m^3^ for water storage). This is multiplied with the size of each subsystem (in m^3^/day for desalination and ZLD, kWh for energy storage, and m^3^ for water storage; values are shown in Table S8) and the amortization factor. A system lifetime n = 30 years is assumed for all subsystems, except for batteries with *n*_*BES*_ = 10 years, at an annual discount rate *r* = 7%.^96^ The amortized annual CAPEX is then averaged over the total freshwater production in a year, which is the product of the desalination *CF* and the combined water production capacity of the desalination subsystem and the ZLD subsystem (when present) for 365 days per year. *CF* of the desalination and ZLD subsystems is 0.25 for configurations with water storage and 1 with energy storage. All input assumptions used are shown in Note S3.

The second term in Equation 1, OPEX_fix_ (in $/m^3^), represents the fixed operations and maintenance expenditures, which are assumed to be 2% of the total system CAPEX divided by the total annual freshwater production, consistent with the literature.^15^^,^^19^^,^^97^ An additional term, OPEX_repl_ (in $/m^3^), accounts for the replacement costs for RO membranes which have a lifetime of 5 years which is less than the system lifetime of 30 years. The next term, OPEX_DWI_ (in $/m^3^), represents the cost of brine disposal through deep-well injection for configurations in which zero-liquid discharge is not pursued.^55^ Finally, OPEX_var_ (in $/m^3^) represents variable operational costs, which is dominated by the cost of energy required for the desalination unit (and ZLD unit when present). This is expressed as the product of the specific energy consumption (SEC) of desalination (and ZLD when present) and the levelized cost of energy: LCOE is used for PV and STE, and LCOH is used for STH. These two parameters will be discussed in greater detail in the following sections.

### Levelized cost of energy

The cost of energy, *i.e.,* OPEX_var_, is one of the main contributors to LCOW. LCOE is a well-established cost metric for electricity generation, which in this analysis includes PV and STE systems (renewable) or CCGTE (fossil fuel baseline). For PV-generated electricity, LCOE depends on the power consumptions of the desalination systems (see Table S7). Power consumptions under 1 MW_e_ are modeled using commercial-scale prices while above 1 MW_e_ the utility-scale price is used. Battery storage is based on lithium-ion batteries, which currently provides over 90% of large-scale electrical storage in the United States.^72^ For STE generation, components include the solar field (with receiver and heat-transfer fluid), power conversion unit, and thermal energy storage (HTTES such as molten salt). In large-scale solar thermal power plants, the energy conversion unit is usually a steam turbine, and the first two components dominate LCOE for typical large-scale concentrated solar power plants that have a capacity over 50 MW_e_.^98^ For distributed desalination, however, the power input ranges from a few hundred kW_e_ (for systems with brine disposal) to a few MW_e_ (for systems with brine concentration). At these smaller capacities, the efficiency of the steam turbine that converts solar-thermal energy into electricity is low, making it impractical for supplying the electrical load of RO, MED, and MVC (when present).^99^ At these scales, a Stirling engine mounted at the focal point of a parabolic dish solar collector is more applicable, with an estimated LCOE of $0.17/kWh_e_ without thermal energy storage.^71^ Here, for all configurations with thermal to electrical energy conversion, LCOW is computed using this LCOE and a thermal storage cost is added assuming a conversion efficiency of 20%.^100^

Unlike LCOE, LCOH is a relatively new metric that compares the economic competitiveness of different thermal energy generation technologies. For this analysis, we consider the LCOH of different solar-thermal systems, which depends strongly on the type of collector used. Broadly, technology options include linear Fresnel collectors, parabolic trough collectors, and power towers or central receiver systems. These tracking systems are designed to achieve temperatures >350 °C for electricity generation or industrial process heat,^101^^,^^102^ making them over-designed for a thermal desalination process (MED requires <90 °C). For lower-temperature applications, stationary collectors including flat plate and evacuated tube collectors, as well as compound parabolic concentrators are better suited. The LCOH is calculated using the equation below:^103^

The CAPEX term includes the total installed cost of the solar collector (in $/m^2^), OPEX_fix_ is the annual fixed operating expenditures (in $/m^2^/yr) which is assumed to be 2% of the CAPEX, and the amortization factor is the same as in Equation 1. The annual thermal-generation capacity is the product of the location-specific solar irradiation (assumed to be 2000 kWh/m^2^/yr, corresponding to a DNI of 6 kWh/m^2^/day)^49^ and the collector efficiency. The LCOH is calculated for different solar-thermal technologies (see Table S6); conventional parabolic troughs have a high LCOH owing to their high CAPEX (*e.g.,* solar field installation cost with optics and tracking, materials and manufacturing cost).^104^ However, new solar collectors designed for temperatures <200 °C (*e.g.,* Artic Solar’s external concentrating parabolic collector, Sunvapor’s “green trough” constructed from wood, and Hyperlight Energy’s plastic linear Fresnel collector) have lower total installed costs.^98^ This translates to a lower LCOH approaching industrial natural gas prices in the United States.^105^ Furthermore, lower-temperature collectors can operate with water-glycol mixtures as the heat transfer fluid and use atmospheric or pressurized hot water as the energy storage medium for LTTES, thereby further reducing the overall desalination system complexity and cost.

## Supplemental information

Document S1. Figures S1–S4, Tables S1–S9, and Note S1–S7

## Footnotes

Supplemental information can be found online at https://doi.org/10.1016/j.isci.2023.105966.
