# Supplementary material for: Distributed desalination using solar energy: A technoeconomic framework to decarbonize nontraditional water treatment
Source: iScience. 2023 Jan 13;26(2):105966. doi: 10.1016/j.isci.2023.105966 (PMC9900398; doi:10.1016/j.isci.2023.105966)
Supplement: Document S1. Figures S1–S4, Tables S1–S9, and Note S1–S7 [file mmc1.pdf]

iScience, Volume *26*

Supplemental information

Distributed desalination using solar energy: A technoeconomic framework to decarbonize nontraditional water treatment

Akanksha K. Menon, Mingxin Jia, Sumanjeet Kaur, Chris Dames, and Ravi S. Prasher

Note S1: Distributed desalination system configurations

There are 16 system configurations being analyzed, and they are referred to as *Configurations 1-16*. Their system compositions and system topologies are shown in Table S1 and Fig. S1, respectively. *Configurations 1 and 2* use PV-generated electricity to drive an RO plant (with BES), and an MED plant (with TES), respectively. In the latter, PV electricity is converted into heat using Joule heating (JH) to satisfy the thermal load of MED, while its electricity input is supplied directly by PV during the daytime and BES at night. *Configurations 3 and 4* use STH to drive an MED plant (with LTTES), and STE to drive an RO plant (with HTTES), respectively. Configuration 3 is further divided into Configurations 3a and 3b: in the former, MED electricity is provided by STE during the day and the associated HTTES is discharged at night, whereas in the latter electricity is provided by PV during the day and by BES at night. With the integration of energy storage, these four configurations run around the clock, *i.e.*, capacity factor = 1.

Configurations 5-8 are similar but use water storage instead, resulting in a capacity factor = 0.25. All 8 systems have limited water recovery and generate brine, which is then disposed by deep-well injection (DWI) at an associated cost (see Table S3). Note that DWI is geographically limited and has a significant environmental impact, which is not accounted for in the disposal cost (*i.e.*, this is an optimistic estimate).

To concentrate the brine to ZLD, *Configurations 9-16* are designed with the same generation-storage-desalination units previously, but now include an MVC unit (powered by either PV or STE). The remaining slurry with a high solids content can be disposed in a landfill at a negligible cost. These 16 system design configurations for solar desalination represent a comprehensive set of commercially relevant technology options.

Table S1: Summary of 16 solar desalination configurations considered in this analysis. Yellow and green highlighting indicates the configurations shown in Table 1 and analyzed in Fig. 4b and 4c, respectively

Configurations	Energy Source			Storage			Desalination		Brine Management	
**Configuration Number and Shorthand**	**Photo-voltaic (PV)**	**Solar-Thermal Heat (STH)**	**Solar-Thermal Electricity (STE)**	**Battery Energy Storage (BES)**	**Thermal Energy Storage (TES)**	**Water Storage (WS)**	**Reverse Osmosis (RO)**	**Multi-Effect Distillation (MED)**	**Deep-Well Injection (DWI)**	**Mechanical Vapor Compression (MVC)**
With Energy Storage and Brine Disposal by DWI										
1: PV + BES + RO + DWI	✓			✓			✓		✓	
2: PV + JH + LTTES/BES+ MED + DWI	✓			✓	✓			✓	✓	
3a: STH/STE (with HTTES) + LTTES + MED + DWI		✓	✓		✓			✓	✓	
3b: STH/PV + LTTES/BES + MED + DWI	✓	✓		✓	✓			✓	✓	
4: STE (with HTTES) + RO + DWI			✓				✓		✓	
With Water Storage and Brine Disposal by DWI										
5: PV + RO + WS + DWI	✓					✓	✓		✓	
6: PV + JH + MED + WS + DWI	✓					✓		✓	✓	
7: STH/PV + MED + WS + DWI	✓	✓				✓		✓	✓	
8: STE (without HTTES) + RO + WS + DWI			✓			✓	✓		✓	
With Energy Storage and Zero-Liquid Discharge (ZLD)										
9: PV + BES + RO + MVC	✓			✓			✓			✓
10: PV + JH + LTTES/BES + MED + MVC	✓			✓	✓			✓		✓
11a: STH/STE (with HTTES) + LTTES + MED + MVC		✓	✓		✓			✓		✓
11b: STH/PV + LTTES/BES + MED + MVC	✓	✓		✓	✓			✓		✓
12: STE (with HTTES) + RO + MVC			✓				✓			✓
With Water Storage and Zero-Liquid Discharge (ZLD)										
13: PV + RO + WS + MVC	✓					✓	✓			✓
14: PV + JH + MED + WS + MVC	✓					✓		✓		✓
15: STH/PV + MED + WS + MVC	✓	✓				✓		✓		✓
16: STE (without HTTES) + RO + WS + MVC			✓			✓	✓			✓

To benchmark these solar desalination systems against state-of-the-art desalination powered by fossil fuels, four baseline system configurations are also analyzed, with their system compositions outlined in Table S2, and their system topologies shown in Fig. S1. The four baselines are: *Baseline 1* - combined cycle gas turbine electricity (CCGTE) is used to drive the RO plant while brine is disposed by DWI; *Baseline 2* - natural gas heat (NGH) from combustion is used to drive an MED plant with electricity supplied by CCGTE and brine disposal by DWI; *Baseline 3* - CCGTE is used to drive an RO plant followed by MVC to achieve ZLD; and *Baseline 4* - NGH is used to drive an MED plant followed by MVC to minimize brine volume. Again, both MED and MVC electricity demands are fulfilled with the same CCGTE unit. The biggest drawback of state-of-the-art desalination is the CO_2 emissions from natural gas; this carbon capture cost is not included in the current analysis and is expected to significantly increase the baseline costs calculated herein.

Table S2: Summary of the four fossil fuel driven desalination configurations (baselines) considered in this analysis. The LCOW of all four baselines are presented in Fig. 4a.

Technology Baselines	Energy Source		Desalination Plant		Brine Management	
	Combined-Cycle Gas Turbine Electricity (CCGTE)	Natural Gas Heat (NGH)	Reverse Osmosis (RO)	Multi-Effect Distillation (MED)	Deep-Well Injection (DWI)	Mechanical Vapor Compression (MVC)
1: CCGTE + RO + DWI	✔		✔		✔	
2: NGH/CCGTE + MED + DWI	✔	✔		✔	✔	
3: CCGTE + RO + MVC	✔		✔			✔
4: NGH/CCGTE + MED + DWI	✔	✔		✔		✔

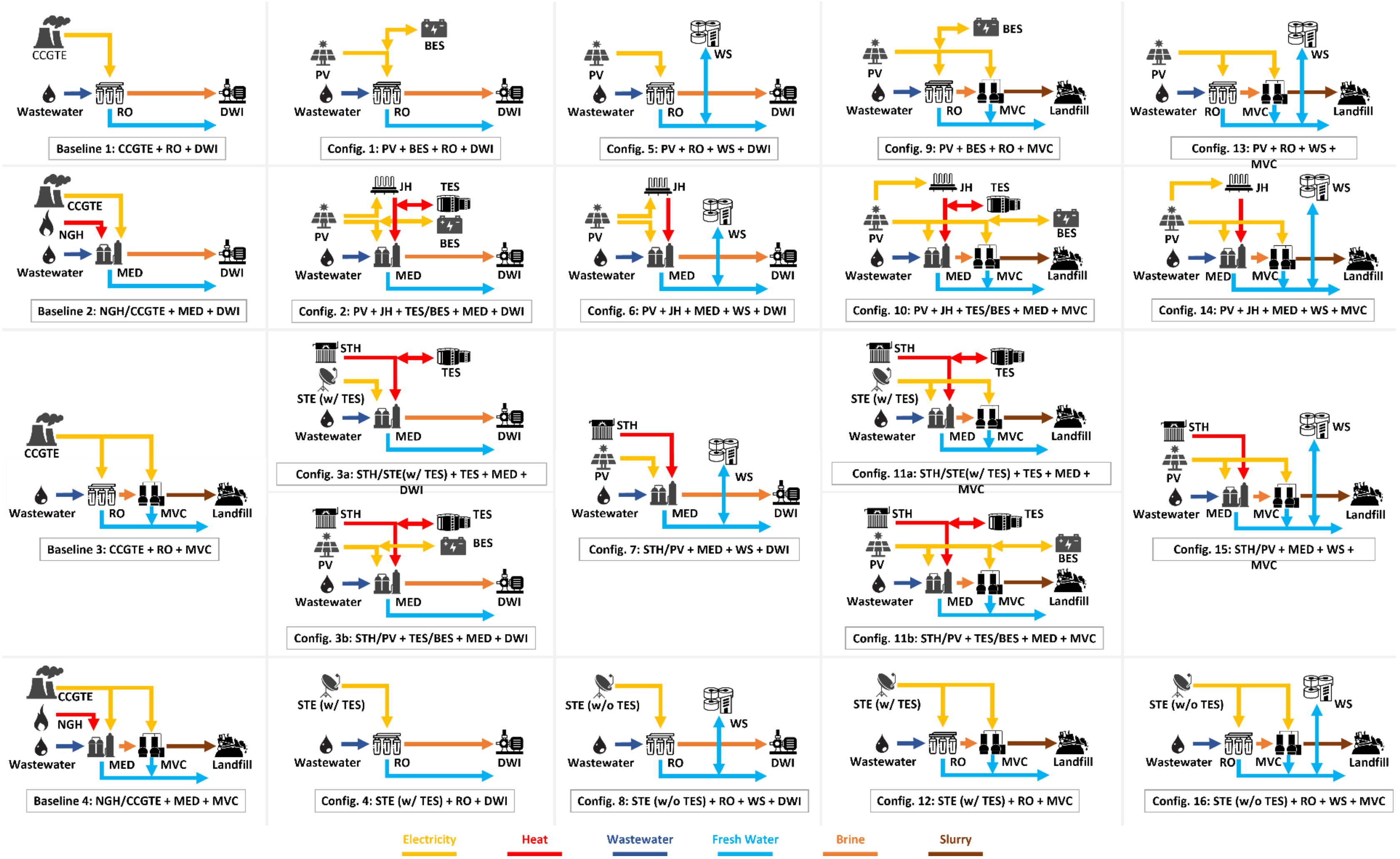

Fig. S1: Summary of system configurations in this analysis. The baseline represents fossil-fuel powered desalination, while configurations 1-16 represent solar desalination systems.

Note S2: Assumptions and Input Variables

Costs and performance data for the different technologies are based on peer-reviewed publications and the GWI DesalData tool. To analyze different systems for distributed desalination at inland facilities, a small-medium scale daily total system capacity ($Size_{total}$) is chosen for the analysis. The daily total system capacity, which is the sum of the desalination capacity and the ZLD capacity (if applicable), is fixed at $Size_{total}$=1000 m^3/day and is assumed to have either energy or water storage. For the energy input, a DNI of 6 kWh/m^2 (annual average) is chosen based on the solar resource available in water-stressed regions (see Figure 1 of the main text). The DNI is the average daily solar energy that can be used for desalination, and in these regions the annually averaged PV capacity factor is ~25%,[1,3,5] which is equivalent to 6 hours of solar input per day. Since the goal is to produce 1000 m^3/day, the desalination plant has to be oversized to meet this demand when operating for a shorter time (only during sunlight hours). We note that hourly TMY data for a specific latitude-longitude can be used to size the desalination system and obtain location-specific cost predictions using the LCOW analysis framework. Other assumptions are that power exchanges with the electric grid are not allowed; and policy-dependent costs (*e.g.,* taxes) and location-dependent costs (*e.g.,* land cost and operator salary) are not included in the analysis. Furthermore, the LCOW values do not reflect the differences in product water quality from RO and MED.

The main terms used to calculate LCOW (see Eq. 1 of the main text) are described in Note S3 by considering the different subsystems. Table S3 lists all the input variables and their values used in this analysis framework.

Table S3: Summary of the input variables used in the high salinity (35,000 mg/L) scenario. The low salinity inputs are shown in Table S9.

Subsystem	Variable	Symbol	Unit	Value	Notes
**Energy**	PV Levelized Cost of Electricity (utility-scale)	$LCOE_{PV}$	$/kWh$_e$	0.046 [2]	In 2017, the solar industry achieved SunShot's original 2020 cost target of $0.06 per kilowatt-hour for utility-scale PV. System capacity is >5 MW$_e$
	PV Levelized Cost of Electricity (commercial)	$LCOE_{PV}$	$/kWh$_e$	0.09 [2]	System capacity is 100 kW$_e$ - 1 MW$_e$
	STE Levelized Cost of Electricity (small-scale)	$LCOE_{ST}$	$/kWh$_e$	0.17 [4]	Small-scale system based on a Stirling dish with capacities ranging from 100 kW$_e$ – 1 MW$_e$. Does not include thermal storage, so this cost is calculated separately.
	STH Levelized Cost of Heat	$LCOH_{ST}$	$/kWh$_t$	0.013	Calculated in Table S6 for low temperature collectors
	Natural Gas Levelized Cost of Electricity	$LCOE_{NG}$	$/kWh$_e$	0.06 [6]	
	Natural Gas Levelized Cost of Heat	$LCOH_{NG}$	$/kWh$_t$	0.01 [7]	Based on 2019 industrial average price for the U.S.
**Desalination**	RO Specific Energy Consumption (High Salinity)	SEC_{RO}	kWh/m^3	5 [8]	RO at smaller capacities does not include energy recovery devices
	MED Specific Energy (Electrical)	$SEC_{MED,el}$	kWh$_e$/m^3	2 [8]	
	MED Specific Energy (Thermal)	$SEC_{MED,th}$	kWh$_t$/m^3	50 [8]	
	RO Capital Cost	$CAPEX_{RO}$	$/(m^3/day)	1200 [9]	
	MED Capital Cost	$CAPEX_{MED}$	$/(m^3/day)	1400 [9]	
	RO Membrane Lifetime	n_{RO}	yr	5 [10]	Membranes are replaced every 5 years at an assumed cost of 10% of $CAPEX_{RO}$.
	Desalination Recovery Ratio	RR_{desal}	%	45 [11]	
**Energy or Water Storage**	Battery Round Trip Efficiency	RTE_{BES}	%	85 [12]	
	Battery Capital Cost	$CAPEX_{BES}$	$/kWh$_e$	390 [13]	Total installed cost for the system including the Li-ion battery
	Battery Depth of Discharge	DoD_{BES}	%	70 [14]	
	Battery Lifetime	n_{BES}	yr	10 [14]	
	Thermal Storage Round Trip Efficiency	RTE_{TES}	%	86 [12]	
	Thermal Storage Capital Cost (High Temperature)	$CAPEX_{TES,H}$	$/kWh$_t$	34 [15]	Molten salt storage total installed cost
	Thermal Storage Capital Cost (Low Temperature)	$CAPEX_{TES,L}$	$/kWh$_t$	17 [16]	Total installed cost of pressurized hot water concrete storage tanks
	Water Storage Capital Cost	$CAPEX_{WS}$	$/m^3	100 [17]	
	Thermal to Electrical Efficiency	η	%	45 [18]	Based on a thermal storage temperature of 400°C
**Brine Management and/or Disposal**	MVC Specific Energy Consumption	SEC_{MVC}	kWh$_e$/m^3	27 [19]	Based on using MVC for brine concentration and not seawater
	MVC Capital Cost	$CAPEX_{MVC}$	$/(m^3/day)	8430 [20]	
	Overall System Recovery Ratio	RR_{sys}	%	95	
	MVC Recovery Ratio	RR_{MVC}	%	91	Calculated based on RR_{desal} and RR_{sys}
	Brine Disposal Cost	C_{DWI}	$/m^3	1.5 [21]	Costs for deep-well injection
**Financial Parameters**	System Lifetime (except battery and membrane)	n	yr	30	
	Fixed Operations and Maintenance Cost	$OPEX_{fix}$	% CAPEX/yr	2 [17,22,23]	
	Discount Rate	r	%/yr	7 [24]	

Note S3: Subsystem size and methodology to calculate LCOW

Desalination and ZLD

1. Capacity Factor (*CF*)

For configurations with water storage but no energy storage (*Configurations 5-8 and 13-16*), the desalination system and, when present, the ZLD system can only operate 6 hours per day. Thus, the capacity factor or desalination utilization is only 0.25. The desalination plant (and the ZLD plant) must be oversized by a factor of 4 to account for this reduced capacity factor. For configurations with energy storage (*Configurations 1-4 and 9-12*), the desalination system and, when present, the ZLD system can operate at full capacity 24 hours per day. Thus, the desalination plant (and the ZLD plant) has a unity capacity factor, and the plant does not have to be oversized.

2. Size of the desalination subsystem ($Size_{desal}$)

Now we consider the sizing of the desalination subsystem. For configurations with energy storage and ZLD (*Configurations 9 - 12*), the daily flowrate of feed water entering the system is given by $\frac{Size_{total}}{RR_{sys}}$, where RR_{sys} is the overall system recovery ratio and is assumed to be 0.95. Then, the capacity of the desalination system (defined as the daily freshwater production rate) can be calculated using Eq. (S1), in which RR_{desal} is the recovery ratio of the desalination subsystem and is assumed to be 0.45. Note that in these configurations, the desalination subsystem capacity is smaller than the total system capacity since it produces only a portion of the freshwater demand, with the ZLD unit supplying the rest.

$$Size_{desal} = Size_{total} \times \frac{RR_{desal}}{RR_{sys}} \tag{S1}$$

For the configurations with water storage and with ZLD (*Configurations 13-16*), the desalination subsystem capacity can be evaluated using Eq. (S2). Note that, as previously mentioned, the oversizing factor $\frac{1}{CF}$ increases the desalination subsystem size and capital cost fourfold compared to the configurations with energy storage, which is a major reason why systems with energy storage turn out to be more economically viable.

$$Size_{desal} = Size_{total} \times \frac{1}{CF} \times \frac{RR_{desal}}{RR_{sys}} \tag{S2}$$

Similarly, sizing the configurations without ZLD follows immediately from Eqs. (S1) and (S2) by setting $RR_{desal} = RR_{sys}$, since in the absence of a ZLD subsystem, all the freshwater is produced by the desalination plant. Specifically, for systems with energy storage and without ZLD (*Configurations 1, 2, 3a, 3b and 4*):

$$Size_{desal} = Size_{total} \tag{S3}$$

And for the configurations with water storage and without ZLD (*Configurations 5, 6, 7 and 8*):

$$Size_{desal} = Size_{total} \times \frac{1}{CF} \tag{S4}$$

3. Size of the zero-liquid discharge subsystem (*Size*$_{ZLD}$)

The size of the ZLD subsystem for configurations with MVC and energy storage (*Configurations 9, 10, 11a, 11b and 12*) is calculated using Eq. (S5). Note that adding the ZLD size calculated here to the desalination subsystem size calculated in Eq. (S1) gives the daily total system capacity.

$$Size_{ZLD} = Size_{total} \times \left(1 - \frac{RR_{desal}}{RR_{sys}}\right) \tag{S5}$$

Similar to the desalination subsystem, the ZLD subsystem is also oversized by a factor of 4× when water storage is used instead of energy storage. The size of such a ZLD subsystem using MVC and water storage (*Configurations 13, 14, 15 and 16*) is calculated by Eq. (S6). Here again, when adding this ZLD size and the desalination size evaluated using Eq. (S2), we get the daily total system capacity multiplied by the oversizing factor.

$$Size_{ZLD} = Size_{total} \times \frac{1}{CF} \times \left(1 - \frac{RR_{desal}}{RR_{sys}}\right) \tag{S6}$$

The remaining configurations (*Configurations 1, 2, 3a, 3b, 4, 5, 6, 7, 8*) accomplish brine disposal by DWI and thus have no ZLD subsystem:

$$Size_{ZLD} = 0 \tag{S7}$$

4. Specific Energy Consumption (*SEC*) of Desalination

The specific energy consumption of the desalination process varies across the different configuration owing to the difference in energy inputs required by electrical and thermal processes. For all configurations that use an MED desalination unit (*Configurations 2, 3a, 3b, 6, 7, 10, 11a, 11b, 14 and 15*), the desalination specific energy is:

$$SEC_{desal,th} = SEC_{MED,th} \quad , \quad SEC_{desal,el} = SEC_{MED,el} \tag{S8}$$

For all other configurations with RO used for water desalination (*Configurations 1, 4, 5, 8, 9, 12, 13 and 16*), the specific energy is:

$$SEC_{desal,th} = 0, \; SEC_{desal,el} = SEC_{RO} \tag{S9}$$

Energy and water storage

5. Size of the low-temperature thermal energy storage subsystem (*Size*$_{LTTES}$)

LTTES is used for storing energy as heat in systems that use STH to drive the MED plant (*Configurations 2, 3a, 3b, 10, 11a and 11b*). The size of the LTTES subsystem, after considering the losses associated with the round-trip efficiency (RTE), should be large enough to meet the thermal load of the desalination subsystem for the entire duration outside the 6-hour solar

window. Therefore, the size of LTTES used to supply thermal energy to the MED subsystem during nighttime is:

$$Size_{LTTES} = (1 - CF) \times \frac{SEC_{desal,th} \times Size_{desal}}{RTE_{TES}} \tag{S10}$$

For all other configurations (*Configurations 1, 4, 5, 6, 7, 8, 9, 12, 13, 14, 15 and 16*), there is no LTTES:

$$Size_{LTTES} = 0 \tag{S11}$$

6. <u>Size of the high-temperature thermal energy storage subsystem (*$Size_{HTTES}$*)</u>

For configurations with STE and energy storage (*Configurations 3a, 4, 11a and 12*), a HTTES subsystem is used to store sufficient thermal energy. This is converted into electricity to drive both the desalination and the ZLD subsystems outside the solar window. In this case, the size of the HTTES is:

$$Size_{HTTES} = (1 - CF) \times \frac{SEC_{desal,el} \times Size_{desal} + SEC_{ZLD} \times Size_{ZLD}}{RTE_{TES} \cdot \eta} \tag{S12}$$

where η = 20%, which corresponds to the corresponding Stirling engine efficiency at a storage temperature is 400 °C. For all other configurations (*Configurations 1, 2, 3b, 5, 6, 7, 8, 9, 10, 11b, 13, 14, 15 and 16*), there is no HTTES:

$$Size_{HTTES} = 0 \tag{S13}$$

7. <u>Size of the battery energy storage subsystem (*$Size_{BES}$*)</u>

For configurations that use BES to store energy (*Configurations 1, 2, 3b, 9, 10 and 11b*), the size of the BES subsystem, after considering the losses associated with the round-trip efficiency, must be such that the stored electricity can drive the desalination and ZLD unit (when present) during the period when solar energy is not available, here taken as 24 × (1-CF) = 18 hours per day. Furthermore, the depth of discharge (*DoD_{BES}*) of batteries must also be accounted for. Thus, the size of the BES subsystem is:

$$Size_{BES} = (1 - CF) \times \frac{SEC_{desal,el} \times Size_{desal} + SEC_{ZLD} \times Size_{ZLD}}{DoD_{BES} \times RTE_{BES}} \tag{S14}$$

For all other configurations (*Configurations 3a, 4, 5, 6, 7, 8, 11a, 12, 13, 14, 15 and 16*) there is no BES:

$$Size_{BES} = 0 \tag{S15}$$

It is also worth noting that in this analysis, the lifetime of the BES subsystem (*n_{BES}*) is assumed to be 10 years rather than to be 30 years, which is the system lifetime. The battery CAPEX is also calculated accordingly based on a 10-year amortization factor. This calculation is reflected in Eq. (1) of the main text.

8. Size of the water storage subsystem ($Size_{WS}$)

For all configurations that use water storage instead of energy storage (*Configurations 5, 6, 7, 8, 13, 14, 15 and 16*), the WS size should be large enough to maintain a constant water supply even when solar energy is not available. The WS size then is:

$$Size_{WS} = (1 - CF) \times Size_{total} \tag{S16}$$

For other configurations (*Configurations 1, 2, 3, 4, 9, 10, 11 and 12*), there is no water storage:

$$Size_{WS} = 0 \tag{S17}$$

Energy Supply

9. Thermal Power from STH (P_{STH})

For configurations that use STH as the energy source and with energy storage (*Configurations 3a, 3b, 11a and 11b*), the generated thermal energy should supply the thermal load of the desalination plant while simultaneously charging the LTTES subsystem during the 6-hour solar window. Thus, the thermal power needed from the solar collector is:

$$P_{STH} = \frac{Size_{LTTES} + SEC_{desal,th} \times Size_{desal} \times CF}{\text{daylight time}} \tag{S18}$$

For configurations using STH and with water storage instead of energy storage (*Configurations 7 and 15*), the collector only needs to satisfy the thermal load of the oversized desalination plant during daylight hours. The excess water produced by the oversized desalination plant is stored in water storge tanks for use during nighttime. Therefore, the thermal power needed from the STH collector in this case is:

$$P_{STH} = \frac{SEC_{desal,th} \times Size_{desal} \times CF}{\text{daylight time}} \tag{S19}$$

For all other configurations (*Configurations 1, 2, 4, 5, 6, 8, 9, 10, 12, 13, 14 and 16*), STH is not used:

$$P_{STH} = 0 \tag{S20}$$

10. Electrical Power from STE (P_{STE})

For configurations using STE and with energy storage (*Configurations 3a, 4, 11a, 12*), the concentrated solar plant needs to supply the electrical load of the desalination subsystem and, when present, drive the ZLD subsystem, while fully charging the HTTES subsystem. The STE power needed is:

$$P_{STE} = \frac{Size_{HTTES} \cdot \eta + (SEC_{desal,el} \times Size_{desal} + SEC_{ZLD} \times Size_{ZLD}) \times CF}{\text{daylight time}} \tag{S21}$$

Here the thermal to electrical energy conversion efficiency, η, is required, since the energy is stored as heat in the HTTES and then converted into electricity using a Stirling engine.

For configurations using STE and water storage (*Configurations 8 and 16*), the STE generation needs to supply the electric load of desalination and, if present, the electrical load for ZLD. Thus, the STE power needed is:

$$P_{STE} = \frac{(SEC_{desal,el} \times Size_{desal} + SEC_{ZLD} \times Size_{ZLD}) \times CF}{\text{daylight time}} \tag{S22}$$

For all other configurations (*Configurations 1, 2, 3b, 5, 6, 7, 9, 10, 11b, 13, 14 and 15*), there is no STE usage:

$$P_{STE} = 0 \tag{S23}$$

11. Electrical Power from PV (P_{PV})

For configurations with PV and energy storage, and when PV is used only to satisfy electrical loads (*Configurations 1, 3b, 9 and 11b*), the power generated during the solar window is used to simultaneously drive the desalination process and the ZLD process (when present), in addition to charging the BES subsystem. The PV electrical power required is:

$$P_{PV} = \frac{Size_{BES} \times \frac{n_{BES}}{n_{sys}} + (SEC_{desal,el} \times Size_{desal} + SEC_{ZLD} \times Size_{ZLD}) \times CF}{\text{daylight time}} \tag{S24}$$

When PV is responsible not only for the electrical loads but also for the desalination thermal load via joule heating (*Configurations 2 and 10*), the PV power is used to simultaneously drive the desalination subsystem (both electrically and thermally), power the ZLD subsystem (when present) and fully charge the BES subsystem. The PV electrical power required then is:

$$P_{PV} = \frac{Size_{BES} \times \frac{n_{BES}}{n_{sys}} + Size_{LTTES} + (SEC_{desal,el} \times Size_{desal} + SEC_{desal,th} \times Size_{desal} + SEC_{ZLD} \times Size_{ZLD}) \times CF}{\text{daylight time}} \tag{S25}$$

Similarly, for configurations with PV and water storage, and when PV is only used for supplying electrical loads (*Configurations 5, 7, 13 and 15*), the PV electrical power needed is:

$$P_{PV} = \frac{(SEC_{desal,el} \times Size_{desal} + SEC_{ZLD} \times Size_{ZLD}) \times CF}{\text{daylight time}} \tag{S26}$$

Likewise, for configurations with PV and water storage, and when PV is powering both the electrical loads and the thermal load (*Configurations 6 and 14*), the electrical power needed is:

$$P_{PV} = \frac{(SEC_{desal,el} \times Size_{desal} + SEC_{desal,th} \times Size_{desal} + SEC_{ZLD} \times Size_{ZLD}) \times CF}{\text{daylight time}} \tag{S27}$$

For all other configurations (*Configurations 3a, 4, 8, 11a, 12 and 16*), there is no PV power generation:

$$P_{PV} = 0 \tag{S28}$$

Please note that all power consumption values in Table S7 are calculated using Eqs. (S18)-(S28).

Operating Costs

12. Fixed operations and maintenance expenditures ($OPEX_{fix}$)

For all configurations, the annual fixed operation cost in $/m^3 is assumed to be 2% of the total system CAPEX, which can be calculated as shown below:

$$OPEX_{fix} = 2\% \times \sum_i \frac{CAPEX_i \times Size_i}{Size_{total} \times 365\ days} \tag{S29}$$

$$i = \text{desal, LTTES, HTTES, BES, WS, ZLD}$$

13. Replacement operation expenditures (*$OPEX_{repl}$*)

The replacement OPEX characterizes the RO membrane replacement cost assuming that its lifetime is n_{RO}. The cost of each replacement is assumed to be 10% of the RO plant CAPEX, which increases the overall LCOW. For configurations using RO as the desalination subsystem (*Configurations 1, 4, 5, 8, 9, 12, 13 and 16*), the operation cost for membrane replacement is:

$$OPEX_{repl} = \frac{\frac{1}{n_{RO}} \times 10\% \times CAPEX_{desal} \times Size_{desal}}{Size_{total} \times 365\ days} \tag{S30}$$

For all other configurations (*Configurations 2, 3a, 3b, 6, 7, 10, 11a, 11b, 14 and 15*), there is no replacement operation cost:

$$OPEX_{repl} = 0 \tag{S31}$$

14. Variable operations cost (*$OPEX_{var}$*)

For all configurations, the variable OPEX is dominated by the cost of energy used in the overall system, which is the product of the amount of energy consumed (*i.e.*, the energy generation calculations above) and the corresponding energy cost (*i.e.*, LCOE or LCOH). Consequently, the variable operations cost is:

$$OPEX_{var} = \frac{(P_{STH} \times LCOH_{ST} + P_{STE} \times LCOE_{ST} + P_{PV} \times LCOE_{PV}) \times \text{daylight time}}{Size_{total}} \tag{S32}$$

15. Brine disposal operations cost using deep-well injection (*$OPEX_{DWI}$*)

The brine disposal OPEX represents the cost associated with deep-well injection in configurations without the ZLD subsystem. For these systems (*Configurations 1, 2, 3a, 3b, 4, 5, 6, 7 and 8*), the brine disposal operation cost is shown in Eq. (S33), where c_{BD} is the per unit brine disposal cost. Note that $OPEX_{DWI}$ is defined as the cost of brine disposal per unit volume of freshwater produced, whereas c_{BD} is defined as the cost of brine disposal per unit volume of brine that is injected underground. The factor $\frac{1-RR_{desal}}{RR_{desal}}$ is for conversion between the two units of measurements based on the two definitions.

$$OPEX_{DWI} = \frac{1-RR_{desal}}{RR_{desal}} \times C_{DWI} \tag{S33}$$

For configurations that achieve ZLD using an MVC unit (*Configurations 9, 10, 11a, 11b, 12, 13, 14, 15 and 16*), the brine disposal cost is zero:

$$OPEX_{DWI} = 0 \tag{S34}$$

Note S4: LCOW for all configurations in the high-salinity scenario

Solar desalination with brine disposal

Starting with a feed salinity of 35,000 mg/L that represents the higher salinity range for nontraditional sources, both RO and MED operate with a 45% water recovery followed by brine disposal *via* deep well injection. Figure S2a shows the eight configurations that fall under this case: for PV-RO (*Configuration 1*), LCOW is higher than fossil-RO (*Baseline 1*), which is primarily due to the high CAPEX of battery storage to ensure continuous operation. Even if the LCOE for utility-scale PV (rather than commercial-scale) is used – which is currently competitive with natural gas prices – the LCOW remains largely unchanged. PV-MED (*Configuration 2*) on the other hand has the highest cost among the cases with energy storage; this is due to MED's high thermal energy consumption, which in this case is provided by electricity for resistive heating. As the price of renewable electricity decreases in the future, the LCOW of this configuration will be lower, but it is unlikely to be economically competitive with other technology options. In fact, even if resistive heating is replaced with a heat pump that has a coefficient of performance of 3, the LCOW reduces to 4.3 $/m^3, which is still higher than many of the other configurations. For STH-MED (*Configuration 3a and 3b*), LCOW is higher than an identical desalination plant driven by natural gas (*Baseline 2*). This can be attributed to the higher current cost of renewable heat even though the cost of energy storage is minimal. Evaluation of the two sub-configurations reveals that STE with high-temperature thermal storage (*Configuration 3a*) is slightly more economical than PV with batteries (*Configuration 3b*) for supplying electricity to MED during nighttime operation. This is due to the lower capital cost of TES compared to BES, which outweighs the lower PV electricity cost compared to STE and makes this one of the most economically attractive technology options. Among all solar-driven configurations, STE-RO (*Configuration 4*) has the lowest LCOW of 3.5 $/m^3, as it combines inexpensive thermal storage with energy-efficient RO desalination. This results in a low storage cost and energy consumption simultaneously, thus yielding a low water cost. Overall, this suggests that solar-thermal driven desalination is more cost-effective than PV-driven desalination for both MED and RO. This is an important finding from a holistic analysis, and it is counter-intuitive from the literature, which is largely dominated by PV-RO where storage costs are not accounted for.

Figure S2a also reveals that desalination with energy storage outperforms systems that store water (configurations 1-4 compared to 5-8). Even though the high cost of energy storage (*e.g.*, capital cost of batteries) is avoided with water storage, this benefit is offset by the higher CAPEX of an oversized desalination plant with a low utilization (capacity factor of 0.25). Furthermore, desalination plants are designed to operate continuously, so the water storage cases are not practical from a technology standpoint even though the storage cost itself is negligible. A similar trend has been observed for PV-RO,[28] and this suggests that investments in energy storage for desalination are necessary.

For the system configurations discussed thus far, a RR of 45% requires brine disposal via deep well injection. In the baseline cases, LCOW values for RO and MED without brine disposal are approximately $1/m^3, which is consistent with literature reports on seawater desalination, [30,32] thereby helping validate the assumptions and analysis framework used herein. The cost of brine disposal, however, increases LCOW by a factor of 3× for the baseline cases and ~1.5-2× for the renewable cases. Furthermore, DWI is permitted only in certain locations and has an adverse environmental impact. These findings highlight one of the major challenges facing nontraditional desalination inland, and it motivates the use of brine concentration technologies that can achieve near-ZLD, thereby significantly reducing the disposal volume and cost.

Solar desalination with zero-liquid discharge

To estimate LCOW for systems with ZLD, we repeat the analysis and include an MVC unit that concentrates the desalination brine to achieve 95% water recovery, and the results are presented in Fig. S2b. For the fossil fuel-driven systems,) as shown in Fig. S2a and S2b, ZLD (*Baselines 3 and 4*) more than doubles the cost of water produced as compared to conventional seawater desalination with 45% recovery and ocean discharge (*Baselines 1 and 2* but ignoring the brown bars for Brine Disposal). However, for distributed desalination, the disposal cost cannot be neglected, in which case the ZLD system (*Baselines 3 and 4*) shows a lower LCOW than the corresponding disposal case (*Baselines 1 and 2* with the brown bars). This suggests that the extra cost of concentrating brine into slurry (which adds MVC capital and energy cost) is offset by the benefits of achieving 95% water recovery and not having to dispose brine through DWI. This is an important finding for existing fossil fuel-driven plants, for which ZLD is economically favorable to inland brine disposal. To the best of our knowledge, this is the first time the advantage of ZLD in quantified for distributed desalination, thereby underscoring the transition to a circular water economy.

ZLD with renewable energy however is currently 1.5× more expensive than the corresponding brine disposal case. For example, in the PV-RO case (*Configuration 9*), the cost of battery storage to power both the RO plant and the MVC unit during hours of low/no solar insolation becomes prohibitively high. PV-MED (*Configuration 10*) is not a viable option for the same reasons as its counterpart (*Configuration 2*), while the use of batteries in *Configuration 11b* makes it economically unattractive as well. *Configurations 11a and 12* which use solar-thermal heat and electricity with thermal storage to drive MED and RO, respectively have the lowest (and comparable) LCOW values, at around 5.2 $/m^3, among all the high water recovery options 9 - 16. The cost of both these configurations is dominated by energy, which is primarily the cost of electricity to drive MVC. The water storage configurations for ZLD (*Configurations 13-16*) are all non-competitive due to the capital cost of the oversized MVC brine concentration system. Here again, solar-thermal driven desalination and ZLD is economically attractive compared to PV-driven processes owing to the lower cost of TES compared to BES for continuous operation.

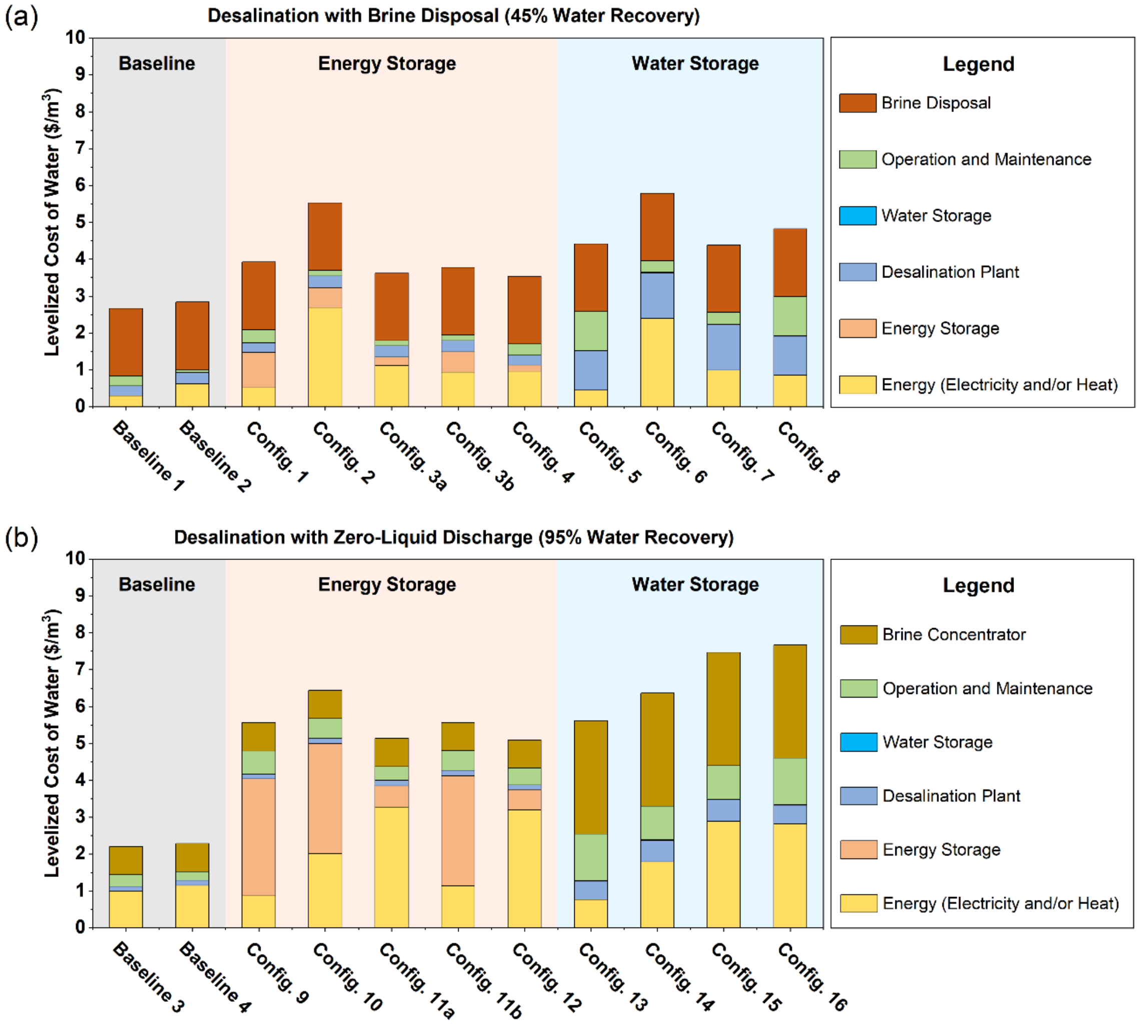

Fig. S2: LCOW for all 16 energy-water system configurations with **(a)** brine disposal after 45% water recovery, and **(b)** ZLD achieving 95% water recovery. In all cases the feed salinity is ~35,000 mg/L (see Fig. 6 for similar analysis of selected configurations for 2,000 mg/L feed). The configurations and baselines are specified in Tables S1, S2 and Fig. S1.

Note S5: Cost projections to 2030

From now to 2030, the cost of renewable energy, energy storage and desalination are expected to decrease significantly. Therefore, a 2030 cost projection is performed to estimate the impact these reduced costs may have on the overall LCOW. The new input parameters used in this analysis are shown in Table S4 below.

Table S4: Current and projected costs of energy, storage, and capital for solar desalination systems

Parameter	Unit	Current Value	2030 Projected Value	Reference
PV Levelized Cost of Electricity (Utility-Scale)	$/kWh_e$	0.046	0.02	[2]
PV Levelized Cost of Electricity (Commercial)	$/kWh_e$	0.09	0.04	[2]
STE Levelized Cost of Electricity	$/kWh_e$	0.17	0.08	[4]
STH Levelized Cost of Heat	$/kWh_t$	0.013	0.008	Assumption
Battery Capital Cost (total installed cost)	$/kWh_e$	309	208	[13]
Thermal Storage Capital Cost (High Temperature)	$/kWh_t$	34	10	[15]
Thermal Storage Capital Cost (Low Temperature)	$/kWh_t$	17	5	Assumption
RO Capital Cost	$/(m^3/day)$	1200	1044	[25]
MED Capital Cost	$/(m^3/day)$	1400	1078	[26]

As shown in Fig. S3, the result of this cost projection analysis indicates that by 2030, the LCOW for solar desalination will drop by a considerable amount. Specifically, desalination systems driven by solar-thermal energy (*Configurations 3a and 4*) with thermal storage will have the lowest LCOW among the cases with brine disposal. This cost is comparable to that of the baseline cases, indicating that renewable desalination is competitive with traditional fossil fuel driven systems. Among the ZLD cases as well, the combination of solar-thermal with thermal storage (*Configurations 11a and 12*) has the lowest LCOW. Although the LCOW is still ~30% higher than the baseline cost, it does not account for the biggest drawback of state-of-the-art desalination, *i.e.*, the CO_2 emissions. The additional carbon capture cost is not included in the current analysis and can significantly increase the baseline costs calculated herein. The analysis predictions for 2030 also suggest that barring unanticipated breakthroughs in battery storage (*e.g.*, significant reductions in Li-ion costs), this form of energy storage is not a good fit for renewable desalination systems.

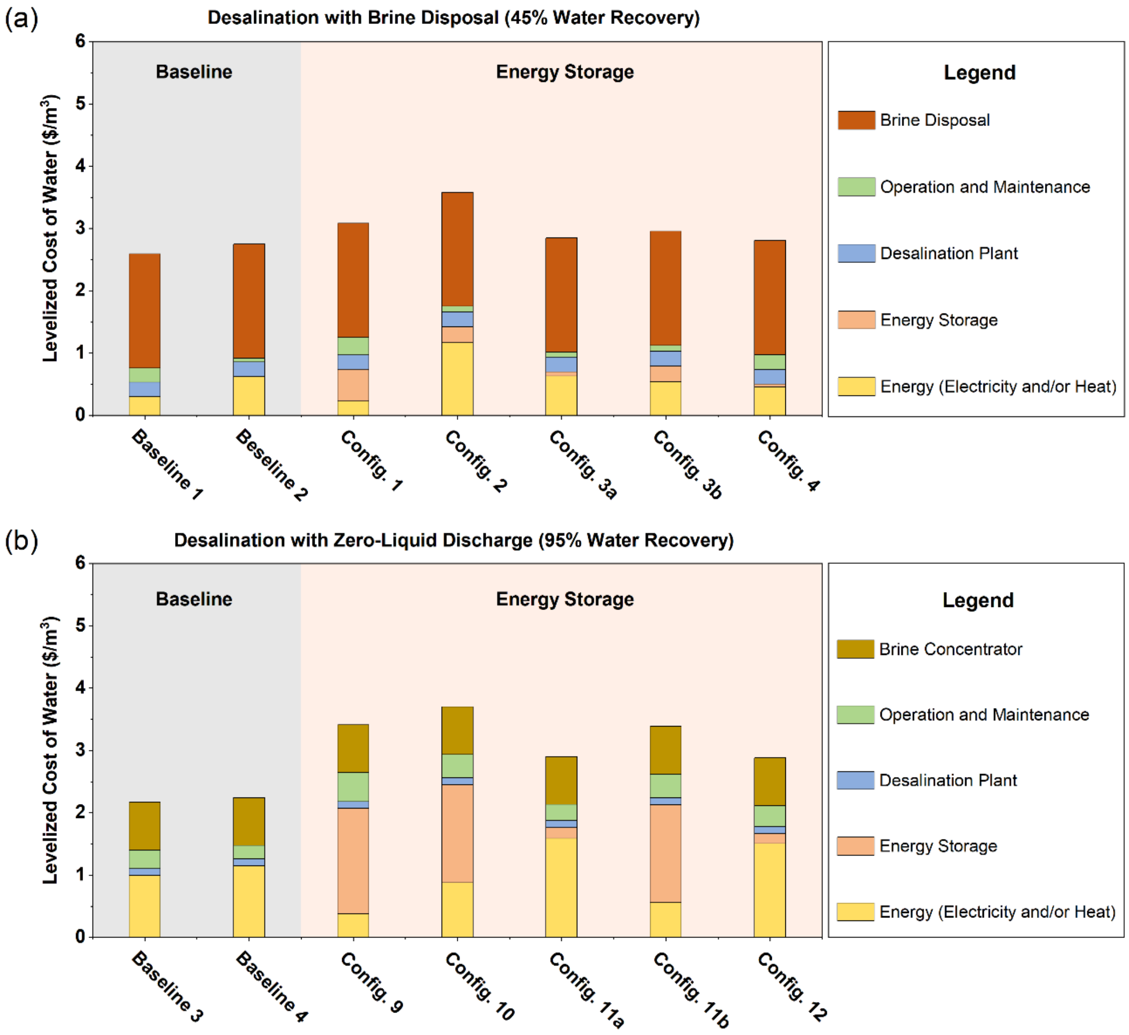

Fig. S3: LCOW projections to 2030 for energy-water system configurations with **(a)** desalination brine disposal after 45% water recovery, and **(b)** ZLD achieving 95% water recovery. The configurations and baselines are specified in Tables S1, S2 and Fig. S1.

Note S6: Single parameter sensitivity analysis

Table S5 shows the ranges used for the sensitivity analysis based on the current input values of the high-salinity scenario. For the performance parameters (top half of the table), some of the ranges are unsymmetric to account for uneven distribution of uncertainties in these variables. For the cost parameters (bottom half of the table), two different ranges are included to account for larger project-specific uncertainties in the values.

Table S5: Single-parameter sensitivity analysis for ZLD system configurations

<table>
<tr><th>Parameter</th><th>Unit</th><th>Low Value</th><th>Base Value</th><th>High Value</th></tr>
<tr><td>RO Membrane Lifetime</td><td>years</td><td colspan="2">5</td><td>10</td></tr>
<tr><td>Recovery Ratio (for RO or MED)</td><td>%</td><td>40</td><td>45</td><td>50</td></tr>
<tr><td>MED Specific Energy (Thermal)</td><td>kWh/m^3</td><td>40</td><td>50</td><td>60</td></tr>
<tr><td>RO Specific Energy (Electricity)</td><td>kWh/m^3</td><td>2</td><td colspan="2">5</td></tr>
<tr><td>System Lifetime</td><td>years</td><td>20</td><td colspan="2">30</td></tr>
<tr><th>Parameter</th><th>Unit</th><th>Low Range</th><th colspan="2">High Range</th></tr>
<tr><td>BES Cost</td><td>$\$/kWh_e$</td><td>$390 \pm 10\%$</td><td colspan="2">$390 \pm 20\%$</td></tr>
<tr><td>TES Cost</td><td>$\$/kWh_t$</td><td>$34 \pm 10\%$</td><td colspan="2">$34 \pm 20\%$</td></tr>
<tr><td>RO CAPEX</td><td>$\$/(m^3/day)$</td><td>$1200 \pm 20\%$</td><td colspan="2">$1200 \pm 30\%$</td></tr>
<tr><td>MED CAPEX</td><td>$\$/(m^3/day)$</td><td>$1400 \pm 20\%$</td><td colspan="2">$1400 \pm 30\%$</td></tr>
<tr><td>PV LCOE</td><td>$\$/kWh_e$</td><td>$0.046 \pm 10\%$</td><td colspan="2">$0.046 \pm 20\%$</td></tr>
<tr><td>STE LCOE</td><td>$\$/kWh_e$</td><td>$0.17 \pm 10\%$</td><td colspan="2">$0.17 \pm 20\%$</td></tr>
<tr><td>STH LCOH</td><td>$\$/kWh_t$</td><td>$0.013 \pm 10\%$</td><td colspan="2">$0.013 \pm 20\%$</td></tr>
</table>

Note S7: LCOW with reduced DWI Cost

In the analysis, we have also explored the impact of reduced DWI cost on the overall LCOW for the configurations with brine disposal rather than with ZLD. As shown in Fig. S4, when the assumed DWI cost is reduced from 1.5 $/m^3$ to 0.5 $/m^3$, the LCOW values of all brine disposal baselines (*Baselines 1 and 2*), and configurations (*Configurations 1-8*) reduce equally by 1.2 $/m^3$. Because of this reduction is the same for all configurations, *Configuration 3a and 4* continue to be the most cost-effective options. However, with the reduction in the baseline LCOW, the two ZLD fossil fuel baselines (*Baseline 3 and 4*) will no longer hold their cost advantage over the brine disposal fossil fuel baselines (*Baselines 1 and 2*).

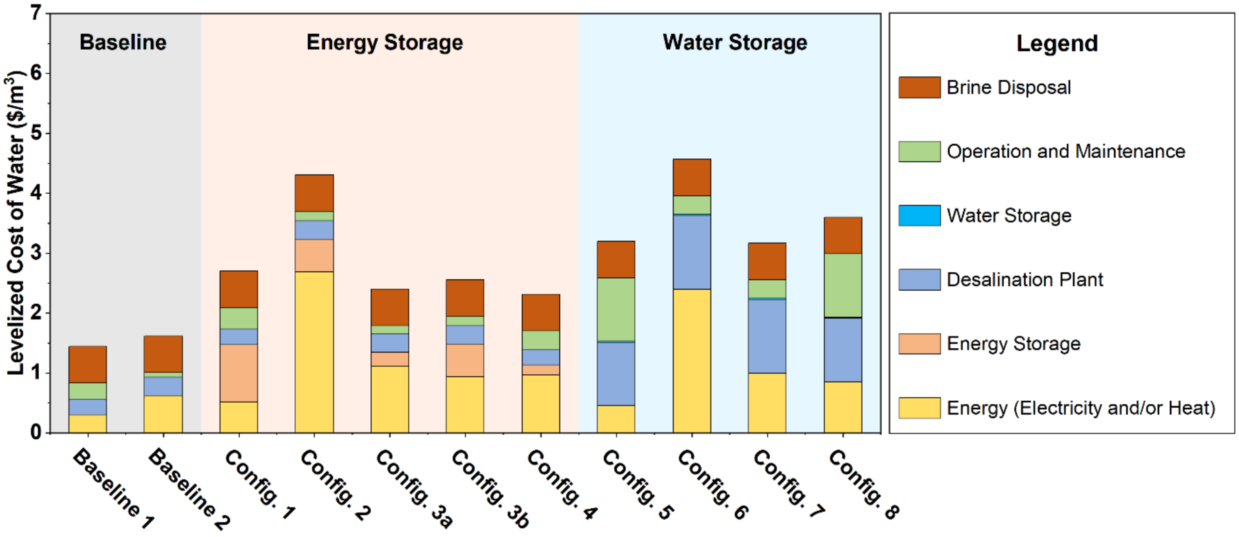

Fig. S4: LCOW of the brine disposal baseline configurations and solar desalination configurations. The DWI cost is reduced from 1.5 $/m^3$ to 0.5 $/m^3$.

Supplemental Tables

Table S6: LCOH comparison of different solar-thermal generation technologies

	Installed cost ($/m^2)	Annual O&M ($/m^2/yr)	Collector Efficiency (%)	Thermal generation (kWh/m^2/yr)	LCOH ($/kWh$_t$)
Conventional parabolic trough collector[27]	300	6	55	1100	0.029
Linear Fresnel collector[27]	240	4.8	35	700	0.036
Low temperature collectors[29]	120	2.4	50	1000	0.013
Natural gas (2019 industrial U.S. average price)[31]					0.010

Table S7: Power consumption using PV, STE and/or STH for all solar desalination configurations for both the high salinity scenario and the low salinity. Multiplying these values by the daylight time (6 hours) yields the daily electrical/thermal energy consumption in kWh_e or kWh_t.

**High Salinity Scenario**		
	Electric Power Consumption (kW_e)	Thermal Power Consumption (kW_t)
**Configuration 1**	944	0
**Configuration 2**	9728	0
**Configuration 3a**	378	9351
**Configuration 3b**	378	9351
**Configuration 4**	946	0
**Configuration 5**	833	0
**Configuration 6**	8667	0
**Configuration 7**	333	8333
**Configuration 8**	833	0
**Configuration 9**	3129	0
**Configuration 10**	7290	0
**Configuration 11a**	2868	4429
**Configuration 11b**	2868	4429
**Configuration 12**	3137	0
**Configuration 13**	2763	0
**Configuration 14**	6474	0
**Configuration 15**	2526	3947
**Configuration 16**	2763	0
**Low Salinity Scenario**		
**Configuration 1**	208	0
**Configuration 4**	208	0
**Configuration 5**	183	0
**Configuration 8**	183	0
**Configuration 9**	438	0
**Configuration 12**	439	0
**Configuration 13**	387	0
**Configuration 16**	387	0

Table S8: Sizes and capacities of the storage, desalination plant and brine management subsystems for both the high salinity scenario and the low salinity scenario

High Salinity Scenario							
	BES Size (kWh_e)	HTTES Size (kWh_t)	LTTES Size (kWh_t)	WS Size (m^3)	Desalination Capacity (m^3/day)	Brine Concentration Capacity (m^3/day)	Brine Disposal Volume (m^3/day)
**Configuration 1**	4412	0	0	0	1000	0	1222
**Configuration 2**	1765	0	43605	0	1000	0	1222
**Configuration 3a**	0	8824	43605	0	1000	0	1222
**Configuration 3b**	1765	0	43605	0	1000	0	1222
**Configuration 4**	0	22059	0	0	1000	0	1222
**Configuration 5**	0	0	0	750	4000	0	1222
**Configuration 6**	0	0	0	750	4000	0	1222
**Configuration 7**	0	0	0	750	4000	0	1222
**Configuration 8**	0	0	0	750	4000	0	1222
**Configuration 9**	14628	0	0	0	474	526	0
**Configuration 10**	13375	0	20655	0	474	526	0
**Configuration 11a**	0	66873	20655	0	474	526	0
**Configuration 11b**	13375	0	20655	0	474	526	0
**Configuration 12**	0	73142	0	0	474	526	0
**Configuration 13**	0	0	0	750	1895	2105	0
**Configuration 14**	0	0	0	750	1895	2105	0
**Configuration 15**	0	0	0	750	1895	2105	0
**Configuration 16**	0	0	0	750	1895	2105	0
**Low Salinity Scenario**							
**Configuration 1**	971	0	0	0	1000	0	53
**Configuration 4**	0	4853	0	0	1000	0	53
**Configuration 5**	0	0	0	750	4000	0	53
**Configuration 8**	0	0	0	750	4000	0	53
**Configuration 9**	2048	0	0	0	953	47	0
**Configuration 12**	0	10240	0	0	953	47	0
**Configuration 13**	0	0	0	750	3811	189	0
**Configuration 16**	0	0	0	750	3811	189	0

Table S9: Differences in input parameters between the low-salinity and high salinity scenarios

Variable	Symbol	High Salinity Scenario	Low Salinity Scenario	Reference
**Desalination Recovery Ratio (%)**	RR_{desal}	45	97	
**Overall System Recovery Ratio (%)**	RR_{sys}	95	99.7	
**RO Specific Energy Consumption (kWh/m^3)**	SEC_{RO}	5	1.1	[33]
**RO Capital Cost ($/(m^3/day))**	$CAPEX_{RO}$	1200	735	[34]

Supplemental References

1. Wu, C., Zhang, X.P., and Sterling, M. (2022). Solar power generation intermittency and aggregation. Sci Rep *12*. 10.1038/s41598-022-05247-2.

2. 2030 Solar Cost Targets | Department of Energy https://www.energy.gov/eere/solar/articles/2030-solar-cost-targets.

3. Chen, S., Lu, X., Miao, Y., Deng, Y., Nielsen, C.P., Elbot, N., Wang, Y., Logan, K.G., McElroy, M.B., and Hao, J. (2019). The Potential of Photovoltaics to Power the Belt and Road Initiative. Joule *3*, 1895–1912. 10.1016/J.JOULE.2019.06.006.

4. Andraka, A.C. (2018). Sandia CSP Dish Technology Assessment.

5. Southwestern states have better solar resources and higher solar PV capacity factors https://www.eia.gov/todayinenergy/detail.php?id=39832.

6. Open Energy Information (2016). Transparent Cost Database. Annu Rev Resour Economics, 1. https://openei.org/apps/TCDB/.

7. EIA expects 2018 and 2019 natural gas prices to remain relatively flat - Today in Energy - U.S. Energy Information Administration (EIA) https://www.eia.gov/todayinenergy/detail.php?id=34672.

8. Al-Karaghouli, A., and Kazmerski, L.L. (2013). Energy consumption and water production cost of conventional and renewable-energy-powered desalination processes. Renewable and Sustainable Energy Reviews *24*, 343–356. 10.1016/j.rser.2012.12.064.

9. Caldera, U., and Breyer, C. (2017). Impact of Battery and Water Storage on the Transition to an Integrated 100% Renewable Energy Power System for Saudi Arabia. Energy Procedia *135*, 126–142. 10.1016/j.egypro.2017.09.496.

10. Sarai Atab, M., Smallbone, A.J., and Roskilly, A.P. (2016). An operational and economic study of a reverse osmosis desalination system for potable water and land irrigation. Desalination *397*, 174–184. 10.1016/j.desal.2016.06.020.

11. Mezher, T., Fath, H., Abbas, Z., and Khaled, A. (2011). Techno-economic assessment and environmental impacts of desalination technologies. Desalination *266*, 263–273. 10.1016/j.desal.2010.08.035.

12. Hameer, S., and van Niekerk, J.L. (2016). Thermodynamic Modelling of Thermal Energy Storage Systems. Energy Procedia *93*, 25–30. 10.1016/j.egypro.2016.07.145.

13. Schmidt, O., Melchior, S., Hawkes, A., and Staffell, I. (2019). Projecting the Future Levelized Cost of Electricity Storage Technologies. Joule *3*, 81–100. 10.1016/j.joule.2018.12.008.

14. McEvoy's Handbook of Photovoltaics (2018). McEvoy's Handbook of Photovoltaics. 10.1016/C2015-0-01840-8.

15. IRENA (2017). Electricity storage and renewables: Costs and markets to 2030.

16. Smallbone, A., Jülch, V., Wardle, R., and Roskilly, A.P. (2017). Levelised Cost of Storage for Pumped Heat Energy Storage in comparison with other energy storage technologies. Energy Convers Manag *152*, 221–228. 10.1016/j.enconman.2017.09.047.

17. Caldera, U., Bogdanov, D., and Breyer, C. (2018). Desalination Costs Using Renewable Energy Technologies. In Renewable Energy Powered Desalination Handbook: Application and Thermodynamics (Elsevier Inc.), pp. 287–329. 10.1016/B978-0-12-815244-7.00008-8.

18. Gheith, R., Aloui, F., and ben Nasrallah, S. (2014). Evaluation of the gamma stirling engine heat transfers in its heat exchangers. In American Society of Mechanical Engineers, Fluids Engineering Division (Publication) FEDSM 10.1115/FEDSM2014-21189.

19. Tong, T., and Elimelech, M. (2016). The Global Rise of Zero Liquid Discharge for Wastewater Management: Drivers, Technologies, and Future Directions. Environ Sci Technol *50*, 6846–6855. 10.1021/acs.est.6b01000.

20. Hisham T. El-Dessouky Hisham M. Ettouney (2002). Fundamentals of Salt Water Desalination 10.1016/b978-0-444-50810-2.x5000-3.

21. Ziolkowska, J.R., and Reyes, R. (2017). Prospects for Desalination in the United States-Experiences From California, Florida, and Texas (Elsevier Inc.) 10.1016/B978-0-12-803237-4.00017-3.

22. Isaka, M. (2012). Water Desalination Using Renewable Energy ENERGY TECHNOLOGY SYSTEMS ANALYSIS PROGRAMME.

23. Cole, W., and Frazier, A.W. (2030). Cost Projections for Utility-Scale Battery Storage: 2020 Update.

24. U.S. DOE (2020). 2020 Discount Rates.

25. Caldera, U., and Breyer, C. (2017). Learning Curve for Seawater Reverse Osmosis Desalination Plants: Capital Cost Trend of the Past, Present, and Future. Water Resour Res *53*, 10523–10538. 10.1002/2017WR021402.

26. Mayor, B. (2020). Unraveling the Historical Economies of Scale and Learning Effects for Desalination Technologies. Water Resour Res *56*. 10.1029/2019WR025841.

27. Blair, N., Diorio, N., Freeman, J., Gilman, P., Janzou, S., Neises, T., and Wagner, M. (2018). System Advisor Model (SAM) General Description (Version 2017.9.5).

28. Ganora, D., Dorati, C., Huld, T.A., Udias, A., and Pistocchi, A. (2019). An assessment of energy storage options for large-scale PV-RO desalination in the extended Mediterranean region. Sci Rep *9*. 10.1038/s41598-019-52582-y.

29. Turchi, C.S., Boyd, M., Kesseli, D., Kurup, P., Mehos, M., Neises, T., Sharan, P., Wagner, M., and Wendelin, T. (2016). CSP Systems Analysis - Final Project Report.

30. Water Desalination Using Renewable Energy: Technology Brief https://www.irena.org/publications/2012/Mar/Water-Desalination-Using-Renewable-Energy.

31. Zaretskaya, V. (2020). Natural gas prices in 2019 were the lowest in the past three years. https://www.eia.gov/todayinenergy/detail.php?id=42455.

32. Ghaffour, N., Missimer, T.M., and Amy, G.L. (2013). Technical review and evaluation of the economics of water desalination: Current and future challenges for better water supply sustainability. Desalination *309*, 197–207. 10.1016/J.DESAL.2012.10.015.

33. Pan, S.Y., Haddad, A.Z., Kumar, A., and Wang, S.W. (2020). Brackish water desalination using reverse osmosis and capacitive deionization at the water-energy nexus. Water Res *183*, 116064. 10.1016/J.WATRES.2020.116064.

34. Triki, Z., Bouaziz, M.N., and Boumaza, M. (2014). Techno-economic feasibility of wind-powered reverse osmosis brackish water desalination systems in southern Algeria. New pub: Balaban *52*, 1745–1760. 10.1080/19443994.2013.807040.
